# Combining Abiotic Stresses as a Low-Cost Strategy for Increasing the Phenolic Content in Apple Agro-Industrial By-Products

**DOI:** 10.3390/antiox14030287

**Published:** 2025-02-27

**Authors:** Esteban Villamil-Galindo, Daniel A. Jacobo-Velázquez, Andrea Marcela Piagentini

**Affiliations:** 1 Instituto de Tecnología de Alimentos, Facultad de Ingeniería Química, Universidad Nacional del Litoral, Santa Fe 3000, Argentina; evillamil@santafe-conicet.gov.ar; 2 Consejo Nacional de Investigaciones Científicas y Técnicas (CONICET), Santa Fe 3000, Argentina; 3 Tecnologico de Monterrey, Escuela de Ingeniería y Ciencias, Av. General Ramón Corona 2514, Zapopan 45201, Mexico; djacobov@tec.mx

**Keywords:** circular economy, natural antioxidant, biofortification, revalorization, UV-A radiation

## Abstract

The circular economy approach offers innovative solutions for valorizing apple by-products through biofortification strategies transforming waste into high-value products and reducing environmental impact. This study evaluates innovative solutions for valorizing Granny Smith apple peel (RM) through biofortification in phenolic compounds using individual or combined abiotic stresses, like wounding stress and ultraviolet A (UVA) radiation. The effects of cutting type (Ct) [whole (C1), 5 mm (C2), 1.5 mm (C3)], storage temperature (ST) [20, 15, 10, 5 °C], and storage time (TM) [0, 12, 24, 48, 72 h] on phenylalanine ammonia-lyase (PAL) and polyphenol oxidase (PPO) activity, total phenolic content (TPC), and phenolic profiles were studied first. The results show that higher stress intensity (C3, 15 °C, 48 h) significantly enhanced secondary metabolism, leading to notable increases in PAL activity (1201%), PPO activity (308%), TPC levels (108–118%), and Procyanidin B2 (PACB2, 22%), the predominant phenolic compound. These changes were critical for improving the bioactive properties and antioxidant potential of RM. The second assay combined wounding stress (same levels of Ct and ST of previous assay, TM: 56 h) with UVA radiation (UVA-D) [0, 86.4 KJ m^−2^, 172.8 KJ m^−2^], determining the optimal conditions (C3, UVA-D 66 KJ m^−2^, 17 °C) for maximizing PAL activity (0.12–0.20 ΔA h⁻^1^ mg⁻^1^), and TPC (3.3 g GAE kg⁻^1^). This study demonstrates the potential of combined abiotic stresses as cost-effective scalable tools to biofortify RM, promoting the sustainable and value-added utilization of agro-industrial by-products.

## 1. Introduction

The agro-industrial sector faces a crucial challenge associated with its rapid expansion. Proper disposal of waste generated during fruit and vegetable processing represents a major challenge for the sector. The apple processing industry is a prime example of the challenges faced by the sector regarding by-product disposal. In 2020, global apple production exceeded 88.2 million metric tons with an estimated value of USD 60.3 billion [1]. Apples are widely consumed worldwide due to their excellent sensory and nutritional characteristics, making them a raw material for various food products like juices, nectars, porridges, sweets, pulp, and soft drinks. The apple processing industry generates significant by-products, including apple peel, core, and seed (6–16% of fruit processed) [2]. Proper disposal of these by-products is crucial to ensure the sustainability of the apple agro-industry sector. Scarano et al. (2022) [3] define these by-products as secondary raw materials with potential applications in numerous industries. These by-products can serve as a source of biofuels, animal feed, organic fertilizers, and functional ingredients like pectin [4,5]. Despite their potential, large quantities of apple by-products remain underutilized and end up in landfills due to the lack of efficient disposal methods.

Generally, plant tissues synthesize secondary metabolites to safeguard the integrity of all their metabolic processes, as these compounds protect the plant tissue from adverse conditions such as excessive solar radiation, reactive oxygen species (ROS), mechanical damage, and cold injury. Researchers have proposed alternative options for valorising agro-industrial apple waste through the extraction of secondary metabolites with bioactive potential, particularly phenolic compounds (PC) [6,7,8]. These compounds exhibit structural diversity with distinct biological properties, particularly in preventing chronic degenerative diseases [9]. The content and profile of apple’s phenolic compounds depend on the variety, agroecological conditions, and apple tissue. Phenolic compounds like chlorogenic acid, catechin, quercetin, rutin, caffeic acid, and pelargonidin can be found in different parts of the fruit [10]. It is well recognized that plants can be subjected to various external stimuli to modulate and increase the synthesis of phenolic compounds, thereby enhancing their bioactive potential through biofortification with phenolic compounds by abiotic stress application [11]. Some of the most extensively studied abiotic stress stimuli include UV radiation (UVC, UVB, and UVA), hyperoxia, hypoxia, hormones such as methyl jasmonate, mechanical damage, or cold damage [12,13]. In the local response, plants immediately synthesize macromolecules such as oligosaccharides and proteins to repair cell damage while simultaneously upregulating the activity of secondary metabolites. Two of the enzymes primarily involved in this response are polyphenol oxidase (PPO, EC 1.14.18.1), which oxidizes phenolic compounds into coloured polymers, causing enzymatic browning and reducing monomeric and polymeric phenol content, and phenylalanine ammonia-lyase (PAL, EC 4.3.1.5), which plays a crucial role in the phenylpropanoid metabolic pathway, promoting the accumulation of phenolic compounds that help the plant regulate the metabolic stress condition and protect against pathogen attack on the vegetal tissue [14]. In the systemic response, the entire tissue is prepared to be more resistant to future attacks, significantly involving an increase in the secondary metabolism with an increment in the activity of PAL and PPO enzymes over time to fulfil this biological function [15]. An increase of 200% in citrus peel by-products’ phenolic compound content was determined after 8 h of storage at 15 °C using dices [16]. Likewise, wounding stress in lettuce produced increases of up to 300% in phenolic compound content after 48 h of storage at 10 °C [17]. Furthermore, Becerra-Moreno et al. [18] reported that wounding stress application in combination with water stress increased total phenolic compound content by 500–1300%.

The intensity of UVA radiation, wounding control, and storage time can affect the accumulation of specific phenolic compounds in plant tissues [19]. Thus, the combined application of UVA radiation and wounding stress as elicitors could be a cost-effective tool to enhance the value of apple by-products as a source of bioactive phenols. A similar approach was applied for strawberry agro-industrial by-products with UVA radiation, increasing anti-inflammatory phenolic compound content [20]. While previous studies have explored the application of individual abiotic stresses, such as UV radiation, wounding, or temperature, to enhance phenolic content in plant tissues, the synergistic effects of combining multiple stressors remain underexplored, particularly in the context of apple agro-industrial by-products. Most research has focused on fresh produce or specific plant species, with limited attention given to the valorization of fruit processing by-products. This approach allows for maximum utilization and revalorisation of these by-products at a low cost by obtaining compounds with high nutraceutical value. Currently, there is no information on the local and systemic response of apple peel to abiotic stress factors. Therefore, the main objective of this work is to study the combined effects of abiotic stress through wounding and UVA radiation, temperature, and storage time on the secondary metabolism and accumulation of phenolic compounds in Granny Smith apple peel. Moreover, the combination of abiotic stresses proposed in this study is designed to be scalable and economically viable for industrial applications. Unlike other methods that require expensive equipment or complex processing steps, this approach utilizes readily available technologies, such as UV lamps and controlled storage conditions, making it accessible to small- and medium-scale agro-industrial operations.

## 2. Materials and Methods

### 2.1. Plant Material

The plant material was the peel of Granny Smith apples (Malus domestica) (RM) obtained from the peeling step of fresh-cut apple processing [2]. The selected apples, free from mechanical or biological damage, were washed with a sodium hypochlorite solution (100 mg L^−1^, pH7) in a 1:3 (*w/v*) ratio for 2 min and rinsed with tap water. Then, apples were cored and peeled with a stainless-steel knife (peel strips 1 mm thick, 20 mm wide, approximately). The apple peel moisture content (80.42 ± 0.52%) was determined in triplicate using a thermogravimetric analyser RADWAG PMR 50 (Poland) at 80 °C for 1 h.

### 2.2. Assay 1: Experimental Design for Biofortification Process Using Wounding-Induced Stress

The abiotic selected conditions were chosen to mimic different post-harvest handling and commercial storage conditions commonly used for fresh-cut and whole fruits. The effect of abiotic stress through wounding damage, temperature, and storage time was evaluated through a general factorial design with three variables: cutting type (Ct) [C1 (whole), C2 (5 mm), C3 (1.5 mm)], storage temperature (ST) (5, 10, 15, 20 °C), and storage time (TM) (0, 12, 24, 48, 72 h). A total of 60 runs were performed in triplicate. After each abiotic stress treatment, samples were frozen at −20 °C until the determination of the activity of the enzymes PAL and PPO, the total phenolic compound content (TPC), and the phenolic compound profile. The effect of the storage time and temperature (*x_i_*) on each experimental response (*Y_k_*) was modelled for each cutting type (Ct) using a second-order polynomial equation (Equation (1)).

(1) $$Y_{k}=\beta_{0}+\sum_{i=1}^{2}\beta_{i}x_{i}+\beta_{i\left(i+1\right)}x_{i}x_{\left(i+1\right)}+\sum_{i=1}^{2}\beta_{ii}x_{i}^{2}$$

where *Y_k_* are the responses (PAL, PPO, TPC, individual phenolic compound content, TPC_HPLC_ (total phenolic content determined by LC); $\beta_{0},\beta_{i},\beta_{i\;(i+1)},$ and $\beta_{ii}$ are the coefficients of the model for each response; and *x_i_* are the independent variables (ST y TM).

The multiple response optimization procedure was performed to determine the Ct, ST, and TM values that maximize PAL, TPC, the major individual phenolic compound content, and TPC_HPLC_ and minimize PPO. The models obtained were validated by comparing the predicted values with the experimental values obtained from an additional experimental run performed at the optimal Ct, ST, and TM values.

### 2.3. Assay 2: Experimental Design for Combined Biofortification Process Using Wounding and UV-A Radiation Stress

The combined effect of wounding stress and UVA-radiation dose to biofortify Granny Smith apple peel (RM) with phenolic compounds was tested following three different types of cuts, C1, C2, and C3, followed by the application of UVA-radiation in a climate chamber (Memmert, Schwabach, Germany) with two 8 W LED-UVA lamps with a 320–400 nm spectrum range, using different doses: control (0 KJ m^−2^), 3 h with 86.4 KJ m^−2^ (UVA3), and 6 h with 172.8 KJ m^−2^ (UVA6). At the half of the radiation time, the RM was turned upside-down to ensure uniform radiation. The samples were then packed in polypropylene trays (30 g) and covered with PVC film. The trays were stored at four different temperatures (20 °C, 15 °C, 10 °C, and 5 °C), and a relative humidity of 90% for a storage time determined on the assay 1 optimization. Subsequently, the samples were frozen at −20 °C and ground to a particle size of less than 1 mm before the analysis.

The activity of the enzymes PAL and PPO were analyzed, along with the total content of phenolic compounds and the individual content of phenolic compounds determined using HPLC-PAD were determined, and the results are expressed as the percentage of variation in the stressed sample (RM-E) (%*VQ*) in reference to no stressed tissue (RM-N), as calculated by Equation (2).

(2) $$\%VQ=\left(\frac{Q_{RM-E}-Q_{RM-N}}{Q_{RM-N}}\right)\times 100$$

where *Q_RM-E_* is the response value of the stressed apple peel (RM-E) and *Q_RM-N_* is the response value of non-stressed apple peel (RM-N) at time 0.

### 2.4. Enzymatic Activity Determinations

#### 2.4.1. Phenylalanine Ammonia-Lyase Enzyme (PAL)

The PAL activity was determined according to Van de Velde et al. [21]. The PAL extract obtained from RM (150 μL) reacts with 1060 μL of Tris–HCl 100 mmol L^−1^ pH = 8.8 and 530 μL of phenylalanine 50 mmol L^−1^ at 37 °C for 1 h. The reaction was stopped by adding 260 μL of TCA 10 g L^−1^. The production of cinnamic acid was measured in a spectrophotometer (Genesys 10 s UV-Vis, Thermo Scientific™, Waltham, MA, USA) at 290 nm. The results are expressed as the absorbance change in one hour per milligram of protein (ΔA/h mg prot).

#### 2.4.2. Polyphenol Oxidase Enzyme (PPO)

The PPO activity was determined according to Van de velde et al. [21]. The PPO-rich extract was obtained by extracting 5 g of RM with 10 mL of extraction solution (phosphate buffer 100 mmol/L pH = 6, PVPP 30 g/L, Triton X-100 0.1% *v/v*, NaCl 1 mol/L), homogenized for 30 s, and stirred for 1 h at 4 °C. The mixture was then centrifuged at 12,000× *g* for 15 min at 4 °C, and the supernatant was collected for further analysis. For the assessment of the activity, 250 μL of extract was added to a reaction mixture containing 900 μL of distilled water, 200 μL of pyrocatechol 200 mmol/L, and 150 μL of phosphate buffer 1 mol/L pH = 6. The mixture was incubated for 1 h at 37 °C, and the absorbance change was measured at 410 nm due to pyrocatechol oxidation. The results are expressed as the absorbance change in one hour per milligram of protein (ΔA/h mg prot).

#### 2.4.3. Protein Content Determination

The protein content of the enzyme extracts was determined using bovine serum albumin as standard. The RM enzyme extracts react with the Lowry reagent (100 parts of 2% Na_2_CO_3_ in NaOH 0.1 M, 1 part of 1% CuSO_4_(5H_2_O), and 1 part of 2% sodium–potassium tartrate) at room temperature for 15 min. Then, Folin–Ciocalteu reagent was added, and after 30 min, the absorbance was read at 680 nm. The protein content in the sample extracts was expressed as milligrams of protein per gram of sample.

### 2.5. Phenolic Compounds Determination

#### 2.5.1. Total Phenolic Content

Total phenolic content (TPC) was determined using the Folin–Ciocalteu method, according to Van de Velde et al. [21]. The extraction of phenolic compounds from RM was performed by mixing frozen ground samples with acetone:water (80:20) (1:5 *w/v*) in two steps. The extracted samples reacted with Folin–Ciocalteu reagent and Na_2_CO_3_ (100 g/L) for 30 min before absorbance was measured at 760 nm (Genesys 10 s UV-Vis, Thermo Scientific™, Waltham, MA, USA). The TPC analysis was carried out in triplicate for each sample, and the results are expressed as grams of gallic acid equivalents (GAE) per kilogram of apple peel (g GAE/Kg).

#### 2.5.2. Phenolic Compound Profile

A LC-20AT high-performance liquid chromatograph with a photodiode array detector (PAD) was used to perform the phenolic compound profile analysis of the RM extracts with a hybrid reverse-phase C18 column Gemini 5μ 110Å of 250 × 4.6 mm attached to a guard column (Phenomenex Inc., Torrance, CA, USA). The Lab Solutions software ver.5.110 was used for data processing and control (Shimadzu Co., Kyoto, Japan). The analysis was conducted according to Villamil-Galindo and Piagentini [22] using a linear gradient of 1% formic acid (A) and acetonitrile (B) as follows: 90–75% of (A) for 30 min, followed by 75–40% of (A) for 30–45 min at a flow rate of 1 mL/min at 25 °C. The identification of the phenolic compounds was tentatively achieved by comparing the retention times and UV-Vis absorption spectra of standard phenolic compounds, as well as previous information obtained using mass spectrometry [23]. The quantification of phenolic compounds was carried out using the external-standard method with the corresponding calibration curves of analytical standards (Sigma-Aldrich Inc., St. Louis, MO, USA), and the phenolic compound concentrations were reported as g/Kg.

### 2.6. Statical Analysis

Tests to verify the assumptions of the ANOVA (normality, independence, and randomness) were performed. The effect of the variables evaluated in the biofortification assay 1 and 2 with phenolic compounds from RM on PAL and PPO activities, TPC, and individual phenolic compound content was determined using analysis of variance (ANOVA). Design Expert software version 8.7.1 was used to create and evaluate the general factorial experimental design through ANOVA analyses and to fit experimental data to the second-order polynomial equations. The linear stepwise regression procedure was used to eliminate nonsignificant terms in each model. Lack of fit and coefficient of determination (*R*^2^) were calculated to verify model adequacy. The optimal abiotic stress conditions were obtained with the multiple response optimization procedure based on the Derringer desirability function. Tukey’s test was used to determine significant differences among abiotic stress treatments, and a *t*-test was run to determine significant differences between the experimental and predicted results to validate the models (*p* < 0.05) using STATGRAPHICS Centurion XV (StatPoint Technologies Inc., Warrenton, VA, USA).

## 3. Results

### 3.1. Assay 1 Biofortification Process Using Wounding-Induced Stress

To ensure practical applicability, the selected types of cutting (Ct), storage temperature (ST), and storage time (TM) were designed to be replicable using widely available food processing equipment, such as commercial food processors with interchangeable blades of varying sizes and cutting intensities and refrigeration chambers and ambient temperatures. The Ct, ST, and TM significantly affected the activities of PAL and PPO enzymes, the total phenolic compound content (TPC and TPC_HPLC_), and the content of procyanidin B2 (PACB2), one of the main phenolic compound of Granny Smith apple peel (RM). The second-order polynomial model fitted the experimental data (R^2^ 0.60–0.82), accurately describing the abiotic stress’ effect on the different responses studied (Supplementary Material, Table S1).

#### 3.1.1. Effect on the Phenylalanine Ammonia-Lyase Activity

**Figure 1:**
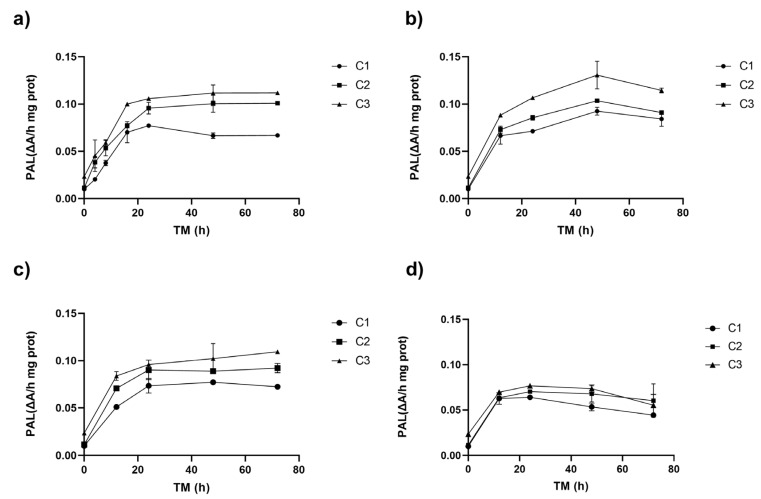
Phenylalanine ammonia lyase enzyme activity (PAL) of apple peel (RM) at different storage times (TMs), cutting types (Cts), and temperatures (STs) of (**a**) 20 °C, (**b**) 15 °C, (**c**) 10 °C, (**d**) and 5 °C. Mean ± standard deviation (*n* = 3). C1: Whole, C2: 6 mm wide, C3: 1.5 mm wide.

Figure 1 shows the PAL activity of RM under different cutting types (Ct), storage temperatures (ST), and storage times (TM). The three experimental variables (Ct, ST, and TM) affected PAL activity (*p* < 0.001). The interaction between ST and TM and the quadratic factors ST^2^ and TM^2^ also affected PAL (*p* < 0.001) (Supplementary Material, Table S1).

The application of different wounding stress intensities as elicitors in the biofortification process with phenolic compounds led to a fast increase in PAL activity after 8–16 h at 20 °C, and after 12 h at 15, 10, and 5 °C (Figure 1). The different cutting types triggered a series of signals that activated the defence mechanism of the RM. The PAL activity in the peel of Granny Smith apples exhibited an immediate dose-dependent response to the intensity of mechanical damage, increasing by 0%, 13%, and 133% for treatments C1, C2, and C3, respectively. Similar results were obtained for potato flesh and peel using wounding stress, with increases of up to 73% and 14% in PAL activity, respectively [24]. The PAL gene expression affecting its activity can vary depending on the plant tissue. Usually, PAL1, PAL2, and PAL3 genes are present and can modulate the synthesis of secondary metabolites that aid in the repair of damaged tissue. [25,26]. The experimental values of the PAL enzyme in RM showed a good fit with the quadratic model, which allows for modelling the behaviour of PAL in RM within the experimental range proposed in this study. PAL activity was modelled for each cutting type showing the maximum activity for C3: 1.5 mm (Equation (3), Figure 1).

(3) $${PAL}_{C3}\left[\frac{\Delta A}{hmgPro}\right]=0.032+5.49E^{-3}\;*\;ST+1.59E^{-3}\;*\;TM+5.04E^{-5}\;*\;ST\;*\;TM-2.42E^{-4}\;*\;{ST}^{2}-2.34E^{-5}\;*\;{TM}^{2}$$

During storage, the cellular metabolism of RM remained active, and a significant increase in PAL was observed for all storage temperatures after 8–16 h of storage, increasing up to 896% for C3 (0.1 ΔA/h mg prot) at 20 °C. The higher PAL activity was obtained at higher wounding stress intensity for all STs (C3, Figure 1). The PAL activity continued increasing during storage, with the maximum value determined after 48–72 h for all experimental assays. However, RM C3 stored at 15 °C exhibited the highest activity after 48 h (0.131 ΔA/h mg prot). This value was 1200% higher than the initial PAL activity of RM (0.01 ΔA/h mg prot, C1, TM 0 h), and 457% higher than C3 (0.02 ΔA/h mg prot, 0 h). Furthermore, the PAL activity of C3 after 48 h was significantly (*p* < 0.05) higher than C2 and C1 (both with similar activities *p* > 0.05, 0.10, and 0.09 ΔA/h mg prot, respectively) (Figure 1b). For the whole apple peel (C1), the maximum increment in PAL activity (669%) was obtained after 48 h at 10 °C. In contrast, for cutting types C2 and C3, the maximum PAL activity increases (818% and 989%, respectively) were obtained after 72 h at 10 °C (Figure 1c). In the RM stored at 5 °C, significant increases occurred during the first 12 h, reaching a value of 0.07 ΔA/h mg prot without significant differences (*p* > 0.05) among Ct, remaining constant during the rest of the storage. The secondary metabolism of RM remains active even at low temperatures; however, the increases in PAL activity are smaller than those determined at higher temperatures (20–15 °C). This finding is critical, as it indicates that the modulation of the secondary metabolism of RM for phenolic compound accumulation requires temperatures above 15 °C. By lowering the storage temperature, the energy requirement for the process is reduced.

Generally, the highest PAL activity occurs around 37 °C [27,28]. Consequently, RM samples subjected to different levels of mechanical damage and stored at lower temperatures (5–10 °C) exhibited less variation over time, with increases ranging from 257 to 690% at 10 °C and 197–537 at 5 °C. This suggests that the PAL enzyme activation in RM follows an intensity-dependent behaviour, corresponding to the severity of the wounding stress applied. These findings align with previous studies on pumpkin, where cutting stress showed a cumulative increase in PAL enzyme activity greater than 100% at greater cutting intensities after 168 h at 4 °C [29]. Similarly, in broccoli, wounding stress triggered phenylpropanoids biosynthesis and enhanced the antioxidant system by increasing the activity of key enzymes and the expression of the related genes. This response significantly boosted phenolic compound accumulation, enhanced free radical scavenging capacity, and improved broccoli’s resistance to wounding [14].

#### 3.1.2. Effect on the Polyphenol Oxidase Activity

The enzyme polyphenol oxidase (PPO) is mainly localized in the chloroplasts of intact plant tissues. When the tissue undergoes mechanical damage, phenolic compounds are released from the vacuole, allowing for the oxidation of o-phenols to o-quinones, which are highly reactive and lead to enzymatic browning [30]. Recent genomic studies have revealed that PPO is involved in several plant defence mechanisms by playing a role in the synthesis of vital precursors in secondary metabolism, like tyrosine and dopamine, acting as an indirect regulator of cell death in plant tissue [31]. The process for obtaining fresh-cut Granny Smith apples involves the mechanical removal of the peel, disrupting plant tissue cell integrity and rendering it more susceptible to the action of the PPO enzyme. Ct, TM, and ST affected PPO activity (*p* < 0.001) in the phenolic compound biofortification process of RM. Additionally, there was a significant interaction between ST and TM that affected the enzyme activity (*p* < 0.01) (Supplementary Material, Table S1). Equation (4) represents the reduced quadratic model that fits properly with the experimental data, predicting the effect of ST and TM on the PPO activity of RM C3.

(4) $${PPO}_{C3}\left[\frac{\Delta A}{h\;mg\;prot}\right]=0.13+9.23E^{-4}\;*\;ST+2.32E^{-3}\;*\;TM-8.74E^{-5}\;*\;ST\;*\;TM-4.96E^{5}\;*\;{ST}^{2}$$

**Figure 2:**
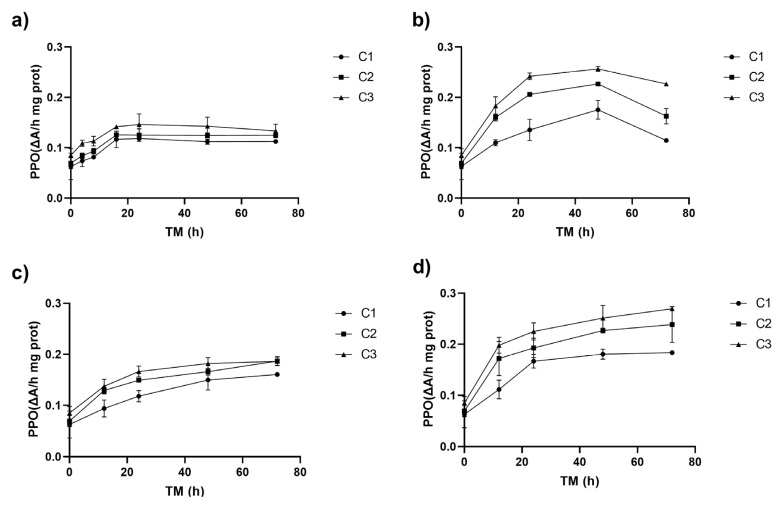
Polyphenol oxidase enzyme activity (PPO) of apple peel (RM) at different storage times (TMs), cutting types (Cts), and storage temperatures (STs) of (**a**) 20 °C, (**b**) 15 °C, (**c**) 10 °C, (**d**) and 5 °C. Mean ± standard deviation (*n* = 3). C1: Whole, C2: 6 mm wide, C3: 1.5 mm wide.

There were no significant differences (*p* > 0.05) among PPO activities due to cutting at TM 0 h (0.06–0.09 ΔA/h mg prot) (Figure 2). Initial PPO activities of Granny Smith apple peel were lower than the PPO values reported for Granny Smith apple flesh (approximately 0.15 ΔA/h mg prot), where the enzyme is more prevalent [32].

The PPO activity significantly increased over the storage time, starting from an initial value of 0.06 ΔA/h mg prot in whole peel (C1 TM = 0). During the first 8–12 h, it rose across all storage temperatures (ST), reaching its peak at 20 °C (up to 132% for C3), and then stabilized (Figure 2a). The highest PPO increases, 261 and 301%, were observed at 15 °C for C2 and C3, respectively, compared to C1 at time 0 (*p* < 0.05), with PPO activities of 0.23 (C2) and 0.26 ΔA/hmg prot (C3) (Figure 2b). Similarly to PAL activity, the higher the Ct intensity, the greater the increment of the PPO enzyme activity. The correlation between PAL and PPO activities was highly significant (*p* < 0.001, R^2^ = 0.66) (Supplementary Material, Table S3).

At 10 °C, PPO activity peaked at 72 h, with increases of 155% for C1 and 197% for C2 and C3. At 5 °C after 72 h, C3 exhibited significantly higher PPO activity (0.27 ΔA/h mg prot), representing a 329% increase compared to C1 at TM = 0 h and a 217% increase compared to C3 at TM = 0 h. PPO activity was influenced by temperature (ST), with significant linear (*p* < 0.001) and quadratic (*p* < 0.01) effects, and a strong interaction with storage time (TM) (Supplementary Material, Table S1). The higher the intensity of the cutting, the higher the activity of PPO, leading to a higher rate of phenolic compound oxidation; the value of this increment will depend on ST and TM values. This reply also triggers the secondary metabolism of the plant, leading to the synthesis of metabolites that help repair the damage caused by the mechanical stress and oxidation processes in the cell lipid membranes [31,33].

#### 3.1.3. Effect on the Total Phenolic Content

**Figure 3:**
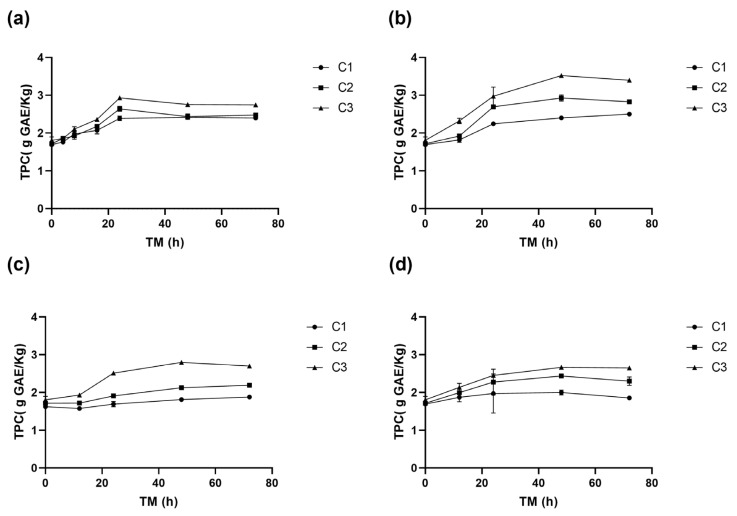
Total phenolic content (TPC) of apple peel (RM) at different storage times (TMs), cutting types (Cts), and storage temperatures (STs) of (**a**) 20 °C, (**b**) 15 °C, (**c**) 10 °C, (**d**) and 5 °C. Mean ± standard deviation (*n* = 3). C1: Whole, C2: 6 mm wide, C3: 1.5 mm wide.

The total phenolic content (TPC) in RM was affected by the three experimental variables (*p* < 0.001) (Supplementary Material, Table S1). A significant TPC increase in was determined during storage, especially in cuts C2 and C3, with the maximum values obtained at 15 °C after 48 h (Figure 3b). Specifically, C3 had the highest TPC (3.5 g GAE/kg), followed by C2 (2.9 g GAE/kg), and the whole peel, C1 (2.4 g GAE/kg). Equation (5) is the reduced model for the TPC change with ST and TM for RM C3.

(5) $${TPC}_{C3}\left(\frac{g\;GAE}{Kg}\right)=1.60+0.022\;*\;ST+0.036\;*\;TM-2.92E^{-4}\;*\;{TM}^{2}$$

These results are consistent with previous studies reporting an increase in TPC in response to mechanical damage [34,35]. Therefore, applying mechanical stress to RM can be a promising strategy to increase its phenolic content, which may contribute to its functional properties and health benefits.

At 20 °C, the TPC of RM increases similarly for the three cutting types until 24 h, when the TPC C3 (2.9 g GAE/Kg) was significantly greater than TPC C2 (2.6 g GAE/Kg RM) and C1 (2.4 g GAE/Kg RM), remaining C3 greater than C2 and C1 up to the end of the storage (Figure 3a). At 15 °C, TPC increased significantly up to 48 h, with an increase of 108% for C3 (3.5 g GAE/Kg) h (1.7 g GAE/Kg), 73% for C2, and 42% for C1, compared to the initial value of C1 at 0 h (Figure 3b).

In wounded RM stored at 10 °C, no significant differences (*p* > 0.05) were observed between TPC of C1 and C2 during storage. However, the TPC of C3 was significantly higher, reaching a maximum value of 2.8 g GAE/Kg at 48 h (Figure 3c). At 5 °C, the increase in TPC was 57% for C3, 44% for C2, and 18% for C1, the TPC of C3 consistently exceeding that of C2 and C1. Similar trends were observed at other storage temperatures, where higher phenolic compound accumulations were associated with greater wounding intensity. A maximum accumulation at 5 °C was recorded at 48 h of storage (Figure 3d).

The highest TPC values were obtained with C3 after 48 h at 15 °C, reaching 3.5 g GAE/Kg. This value is similar to those reported by Guyot et al. [36] for Granny Smith apple peel (3.1 g GAE/Kg), but higher than Granny Smith flesh (0.5–0.9 g GAE/Kg) reported by Serra et al. [32]. The TPC in RM increased rapidly during the first 8–12 h of storage at different temperatures, particularly for higher cutting intensities (C2 and C3), similar to PAL and PPO activities (Figure 1, Figure 2 and Figure 3). The highest TPC value was determined after 24–48 h of storage, significantly correlating with the PAL and PPO enzymes activities (*p* < 0.001, R^2^ 0.77 and 0.70, respectively) (Supplementary Material, Table S3). This suggests that a greater wounding stress intensity strongly activates the RM secondary metabolism, where the synthesis rate of phenolic compounds surpasses their oxidation rate, leading to their accumulation. A similar response has been observed in fresh-cut potatoes, where cutting induced phenylalanine ammonia-lyase (PAL) activity and significantly enhanced phenol content by 40.48%, 74.88%, and 108.86% in pieces, strips, and slices, respectively. Additionally, this response was accompanied by an increase in the activity of antioxidant enzymes, including superoxide dismutase, catalase, ascorbate peroxidase, and glutathione reductase, which contributed to a 1.37–1.46-fold increase in total antioxidant capacity compared to the control [37].

#### 3.1.4. Effect on the Phenolic Compound Profile

Apple are widely consumed worldwide due to their nutritional, organoleptic, and bioactive properties. Their frequent consumption has been recommended to prevent various health conditions [38]. These bioactive properties are attributed to phenolic compounds, which vary based on endogenous factors, such as variety and genotype, and exogenous conditions such as agro-ecological production practises [39]. In the case of RM, this tissue by-product of the agro-industry is currently utilized primarily for pectin extraction but remains underutilized as a potential source of nutraceutical compounds [40]. This study identified ten phenolic compounds in RM from five classes: flavan-3-ols with (+) catechin ((+)CTQN), (-) epicatechin ((-)EPQN); proanthocyanidins with procyanidin B2 (PACB2) and procyanidin tetramer (PACT); phenolic acids with chlorogenic acid (ACl); flavonols with quercetin-3-0-glucuronide (Q3G), quercetin pentoxide (QP), quercetin hexoside (QHS), and kaempferol-3-o-glucuronide (K3G); and, finally, dihydrochalcones with phloretin (FLN).

**Figure 4:**
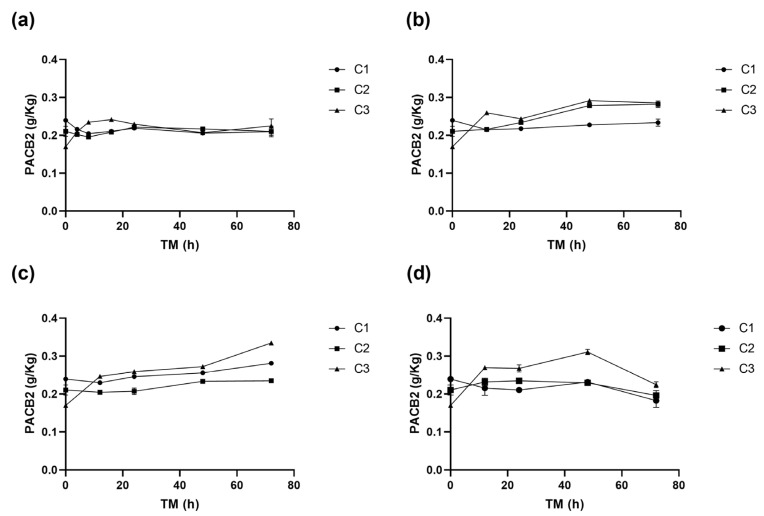
Procyanidin B2 (PACB2) concentration of apple peel (RM) at different times (TMs), cutting types (Cts), and storage temperatures (STs) of (**a**) 20 °C, (**b**) 15 °C, (**c**) 10 °C. (**d**), and 5 °C. Mean ± standard deviation (*n* = 3). C1: Whole, C2: 6 mm wide, C3: 1.5 mm wide.

Table 1, Table 2, Table 3 and Table 4 show the changes in the concentrations of the individual phenolic compounds for different cutting types (Cts), temperatures (STs), and times (TMs). The initial (+)CTQN concentration was 0.02 g/Kg (C1 TM = 0 h). The biofortification process produced higher increases in concentrations after 72 h at 15 °C, 332%, 356%, and 314% for C1, C2, and C3, respectively (Table 2). PACB2 was the major phenolic compound in RM, with a content of 0.24 g/Kg, which was significantly affected (*p* < 0.05) by Ct, ST, and TM, as well as by the interactions between Ct and TM (*p* < 0.01) and their quadratic terms (Supplementary Material, Table S1). The wounding stress immediately (0 h) reduced PACB2 concentration by up to 28% for C3 (Figure 4), possibly due to its susceptibility to oxidation by the PPO enzyme. According to the Pearson correlation analysis (Supplementary Material, Table S3), PACB2 had a highly significant correlation (*p* < 0.001) with PPO enzyme activity (R^2^ 0.5). However, during storage, PACB2 concentration increased to 0.31 g/kg at 5 °C (Table 4) and 0.28 g/Kg at 15 °C (Table 2). These concentrations of procyanidin B2 were higher than those reported for Granny Smith apple flesh (0.1 g/Kg) and ’Golden’ apple peel (0.08 g/Kg) [41]. The experimental PACB2 concentrations for C3 have been appropriately fitted to Equation (6).

(6) $${PACB2}_{C3}(\frac{g}{Kg})=0.17+8.81E^{-3}ST+2.55E^{3}\;*\;TM-3.24E^{5}\;*\;ST\;*\;TM-3.93E^{-4}\;*\;{ST}^{2}-1.54E^{-5}*{TM}^{2}$$

The (-)EPQN exhibited a maximum increase of 60% in RM subjected toC3 and stored at 15 °C for 48 h, rising from 0.1 to 0.16 g/Kg RM. Similarly, PACT concentration increased by up to 156% under the same conditions (Table 2). Flavonols were the second class of phenolic compounds present in RM, and these secondary metabolites were the only ones that showed a significant immediate effect after subjecting RM to wounding stress (Table 1, Table 2, Table 3 and Table 4).

The Q3G initially had an average concentration of 0.04 g/Kg of RM, which significantly increased (*p* < 0.05) by 23% for C2 (0.047 g/Kg RM) and 40% for C3 (0.054 g/Kg). The highest accumulation was recorded at 0.18 g/Kg (370% higher than C1 TM = 0) after 48 h of storage at 15 °C (Table 2). A highly significant correlation was found between Q3G and PAL activity (*p* < 0.001 R^2^ 0.72), suggesting that quercetin is modulated as one of the most important metabolites of the systemic response in RM for defending against the imposed abiotic stress condition. Similarly, C3 generated an immediate increase in QP by 232%, with an increase of 630% (from 0.01 g/Kg RM to 0.19 g/Kg RM) after 24 h at 15 °C. A comparable response has been observed in forced chicory root by-products. Studies demonstrate that wounding and water loss treatments significantly increase caffeoylquinic acids content, with chlorogenic acid levels rising 3.3-fold after wounding and 244 h of drying at room temperature, while dicaffeoylquinic acids increased 2.3-fold after wounding and 71 h of drying at room temperature. This biofortification mechanisms are highly dependent on temperature and water content, emphasizing the complexity of phenolic compound regulation in response to abiotic stress [42].

For QHS and K3G, C3 also produced maximum increases of 307% and 220%, respectively, after 48 h at 15 °C. FLN, a chalcone, significantly increased (845%) with C3 after 48 h at 15 °C, indicating its significant participation in the systemic response of RM, as chalcones play different biological functions in plants such as fertility, pigmentation, pollination, and growth regulation [43].

**Figure 5:**
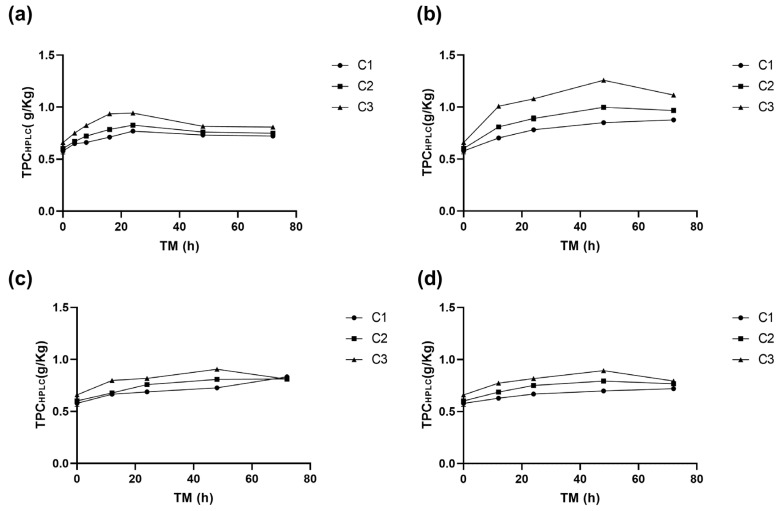
Total phenolic content of apple peel (RM) determined using HPLC (TPC_HPLC_) at different times (TMs), cutting types (Cts), and storage temperatures (STs) of (**a**) 20 °C, (**b**) 15 °C, (**c**) 10 °C, (**d**) and 5 °C. Mean ± standard deviation (n = 3). C1: Whole, C2: 6 mm wide, C3: 1.5 mm wide.

The total of identified individual phenolic compounds (TPC_HPLC_) was significantly affected by cutting intensity (Ct), storage temperature (ST), and time of storage (TM) (*p* < 0.001), as well as by the quadratic terms of ST and TM (*p* < 0.01) (Supplementary Material, Table S1). The application of the highest intensity wounding damage (C3) immediately increased TPC_HPLC_ by 14.2% compared to C1, indicating a significant effect (*p* < 0.05). This trend was similar to the one determined for (+)CTQN, PACT, Q3G, and QHS. However, the highest phenolic compound accumulation in RM was found at 15 °C. Throughout the storage period, the trend of higher TPC_HPLC_ content for C3 was maintained, with a significant increase of 118% (1.26 g/Kg) at 48 h, followed by C2 with 73% (1 g/Kg), and finally the whole peel C1 with 47% (0.85 g/Kg). The values obtained for biofortified RM with C3 at 15 °C and 48 h were 35%, 28%, and 29% higher than those obtained for C3 after 48 h at 20 °C, 10 °C, and 5 °C, respectively (Figure 5). These results suggest that storing C3 at 15 °C favours the secondary metabolism of RM. The reduced quadratic model for predicting TPC_HPLC_ in C3 as a function of storage time (TM) and storage temperature (ST) is presented in the following Equation (7).

(7) $${TPC}_{HPLC-C3}\left(\frac{g}{Kg}\right)=0.48+0.04\;*\;TM+9.85E^{-3}\;*\;ST-1.37E^{-3}\;*\;{ST}^{2}-9.97E^{-5}E-005\;*\;{TM}^{2}$$

These biofortification conditions with phenolic compounds in RM show a significant correlation with the activity of PAL and PPO enzymes, as well as TPC determined using the Folin–Ciocalteu methodology (*p* < 0.001, R^2^ 0.80, 0.72, and 0.87, respectively) (Supplementary Material Table S3). Therefore, using different intensities of wounds, storage temperatures, and times is a feasible, safe, simple, and efficient tool to modulate the synthesis of phenolic compounds in agro-industrial by-products of Granny Smith apples.

#### 3.1.5. Optimum Conditions of RM Biofortification Process Using Wounding-Induced Stress (Assay 1)

The data obtained from the main studied responses (PAL, PPO, TPC, PACB2, TPC_HPLC_) in the RM biofortification process were adequately fitted to quadratic equations (Equations (3)–(7)). Optimization of multiple responses was performed to determine the ST, TM, and Ct values that maximize PAL, TPC, PACB2, and TPC_HPLC,_ while minimizing PPO. The optimum variables for the RM biofortification process (desirability 0.72) were C3, ST: 17 °C, and TM: 56 h.

Under optimal conditions, the equations of the RM biofortification process were experimentally validated. For PAL activity, the predicted value was significantly higher (0.12 ΔA/hmg prot, *p* < 0.05) than the experimental one (0.09 ΔA/hmg prot). This value was higher than was determined initially in RM, indicating the activation of the secondary metabolism of RM due to wounding and storage stress. Regarding PPO activity, the experimental value was (0.195 ΔA/hmg prot) significantly higher than the predicted one (0.18 ΔA/hmg protein). The experimental value of PACB2 (0.28 g/Kg) was significantly higher (*p* < 0.05) than the predicted one (0.26 g/Kg). This is a positive result, considering that the goal is to achieve the greatest accumulation of compounds with high bioactive potential. Regarding TPC and TPC_HPLC_, there were no significant differences between the predicted values (3.0 g GAE/Kg, 1.0 g/Kg RM, respectively) and experimental ones (2.7 g GAE/Kg,0.90 g/Kg RM, respectively), validating the mathematical models obtained.

### 3.2. Assay 2: Combined Biofortification Process Using Wounding and UV-A Radiation Stress

#### 3.2.1. Effect on PAL Enzyme Activity

The type of wounding type (Ct), storage temperature (ST), and UVA dose (UVA-D) significantly affected PAL activity (*p* < 0.01) in RM samples stored for 56 h (optimal storage time determined in the assay 1 optimization). Similarly, both ST and UVA-D had a significant effect on PAL activity (*p* < 0.001) (Supplementary Table S2). Exposure to UVA radiation triggered an immediate response in PAL activity, increasing by 110% and 142% after 3 h of radiation (UVA3) and from 44% to 148% after 6 h of radiation (UVA6) (Table 5). However, for both UVA doses, no significant differences (*p* > 0.05) were observed among the different cutting treatments.

After 56 h of storage at 20 °C following UVA6 radiation, reduced PAL activity was shown, with reductions of up to 58% for C1. Conversely, at 15 °C, increases in PAL activity of up to 193% were determined for C3 UVA3 (0.11 ΔA h^−1^ mg^−1^), which was significantly higher (*p* < 0.05) than the activity of C1 and C2 (0.07–0.09 ΔA h^−1^ mg^−1^), and higher than the obtained values for UVA6 under these same conditions, and higher than the UVA3 sample stored for 53 h at 10 and 5 °C. These findings indicate a delayed yet progressive activation of the secondary metabolism of RM after cutting and UVA3 radiation, similar to the results reported for blueberries irradiated with UV-B, where the expression of PAL-related genes peaked at 24 h [44].

#### 3.2.2. Effect on PPO Enzyme Activity

The results show that Ct stress and UVA-D significantly (*p* < 0.05) affected the PPO activity of RM, as well as the interaction between ST and UVA-D (Supplementary Material Table S2). At the beginning of the experiment (time 0), the PPO activity of the whole RM was 0.08 ΔA h^−1^ mg^−1^. Immediately after UVA radiation, the PPO activity for C2 increased to 0.14 ΔA h^−1^ mg^−1^ with UVA3 and 0.12 ΔA h^−1^ mg^−1^ with UVA6. The late effect of the RM biofortification elicitors showed that storage at 20 °C with UVA3 favoured PPO activity (Table 5), with increases of up to 470% for the different Cts, with no significant differences (*p* > 0.05) among them. The increases in PPO activity for UVA6 at 20 °C ranged 112–156%, with no differences among Ct. In the experiments conducted on RM, no significant differences (*p* > 0.05) were determined between the UVA3 and UVA6 treatments for each type of cutting stored at 15, 10, and 5 °C. However, the results show that the elicitors studied activated the secondary metabolism of RM, as evidenced by the increase in PAL activity, which showed a weak significant correlation with PPO enzyme activity (*p* < 0.05 R^2^ 0.26) (Supplementary Material Table S4) The results show that at higher STs, PPO activity is greater, regardless of the cut. However, at 20 °C, PPO activity decreases with higher doses of UVA due to the inhibitory effect of this radiation on PPO. The use of UV-A LED radiation (390 nm) as an eco-friendly, safe, and low-cost alternative has been reported for the control of enzymatic browning of fresh-cut Granny Smith apple flesh irradiated for 60 min [45]. However, its effect on Granny Smith apple peel and its synergistic effect with wounding stress as elicitors to biofortify this agro-industrial waste with phenolic compounds has no previous precedent.

#### 3.2.3. Effect on Total Phenolic Compounds

RM can be considered a biofactory of phenolic compounds, and its metabolic capacity can be exploited. Therefore, the Ct, UVA-D, and the different STs during 56 h affected (*p* < 0.01) the TPC. Furthermore, the quadratic term of UVA-D also affected (*p* < 0.05) the recovery of TPC (Supplementary Material Table S2).

Immediately after wounding stress, RM showed an increase in TPC, with C1 at 1.10 g GAE kg^−1^, C2 at 1.31 g GAE kg^−1^, and C3 at 1.46 g GAE kg^−1^. When subjected to UVA-D, significant increases in TPC accumulation were obtained. Among the UVA-D treatments, UVA3 had the highest increase, with C1 at 104% higher than C2 and C3 (76% and 86%, respectively) compared to the same cutting type without radiation. However, for all Cts with UVA3, TPC was higher than UVA6, considering that UVA6 samples had an initial TPC increase in only 22% for C1 and 12% for C3, and a 2.6% TPC reduction for C2. Similarly to PAL and PPO activity, the highest phenolic compound accumulation was obtained for biofortified tissue of RM C3 UVA3 stored during 56 h at 15 °C, which correlated with PAL and PPO (R^2^ 0.73, 0.35, respectively). In those conditions, the highest increase (*p* < 0.05) in TPC was 174% (4.0 g GAE kg^−1^), being significantly higher than TPC for UVA6 (2.06 g GAE kg^−1^). The results obtained in this study demonstrate a synergistic effect between cutting stress and UVA radiation, leading to a higher concentration of phenolics even compared to the influence of wounding stress alone. This change was further enhanced during storage. The results were higher than those reported for red cactus skin, where the combined effects of UV-B stress and wounding did not immediately affect TPC. However, it showed a delayed effect after 24 h of storage, with a maximum increase of 33.8% with a UV-B radiation time of 15 min [46].

#### 3.2.4. Effect on Individual Phenolic Compounds

**Figure 6:**
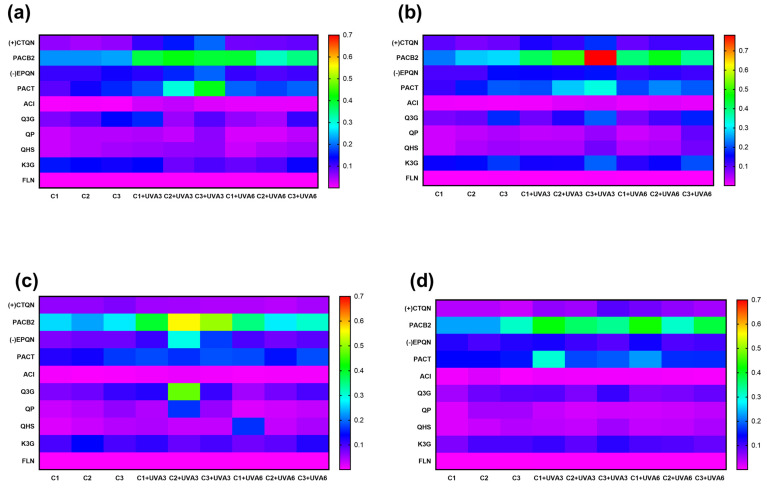
Heatmap visualization of individual phenolic compound concentrations of apple peel (RM) at different abiotic stress treatments. UVA3: 86.4 KJ m^−2^; UVA6: 172.8 KJ m^−2^; C1: Whole M. C2: 5 mm wide, C3: 1.5 mm wide. (+) CTQN: (+) Catechin; PACB2: Procyanidin B2; (-)EPQN: (-) Epicatechin; PACT: Procyanidin tetramer; ACL: Chlorogenic acid; Q3G: Quercetin-3-o-glucuronide; QPN; Quercetin pentoxide; QHS: Quercetin hexoxide; K3G; Kaempferol-3-o-glucuronide. TPC_HPLC_: Total phenolic content quantified using HPLC. At (**a**) 20 °C. (**b**) 15 °C. (**c**) 10 °C. (**d**) 5 °C.

The biofortification process variables (Ct, ST, and UVA-D) did not affect the individual phenolic compounds determined in RM, but did affect their concentrations (Figure 6). UVA radiation modulates photosynthesis processes in plants and has been shown to have an impact on certain individual secondary metabolites. For RM, the concentration of the main phenolic compound, PACB2, was significantly improved by UVA radiation and wounding stress. At RM, PACB2 was tentatively identified as the major phenolic compound of RM, with an initial concentration of 0.2 g Kg^−1^. This phenolic compound was affected by Ct, UVA-D (*p* < 0.01), and ST (*p* < 0.05), as well as the interaction between Ct and UVA-D, which suggests that both wounding stress and UVA radiation synergistically contribute to its increase, specifically at 15 °C after 56 h. Immediately after exposure to UVA-D, significant increases were determined for samples UVA3 and UVA6, reaching an average concentration of 0.78 g Kg^−1^ for sample C3 (Supplementary Material, Figure S1). This concentration was 305% higher than C3 without initial radiation and 15% higher than C3 after exposure to UVA3. Furthermore, it was significantly higher (*p* < 0.05) than C3 exposed to UVA6 (0.37 g Kg^−1^).

The biofortification process also affected the concentrations of other phenolic compounds (Figure 6). (+)CTQN exhibited a maximum increase of 209% under C3 and UVA3 at 20 °C (0.2 g Kg^−1^), showing a significant correlation (*p* < 0.001 R^2^ 0.57) with PPO activity, suggesting that it may be a key substrate of this enzyme during the biofortification process in this study (Supplementary Material, Table S4). (-)EPQN showed its maximum increase with C2 at 10 °C, with UVA3 being 173% higher than the initial C1. PACT increased up to 121% with C3 and UVA3 at 20 °C, and ACl increased by 231% with C3 and UVA3 at 15 °C.

Q3G increased by 202% for UVA3 (0.2 g Kg^−1^) and by 150% for UVA6 (0.17 g Kg^−1^) without significant differences (*p* > 0.05). Otherwise, PACT and PACB2 were the individual phenolic compounds with the highest correlations with PAL (R2: 0.50 and 0.49, respectively). QP and QHS, with C3 at 15 °C, increased by 140% for both UVA radiation doses (86.4 KJ m^−2^ and 172.8 KJ m^−2^). UVA3 irradiation, with C3 and 15 °C of ST, produced an increment of 106% and 142% for K3G and FLN, respectively. These results demonstrate that the synthesis of most phenolic compounds increases with a higher wound intensity, accompanied by a radiation dose of 86.4 KJ m^−2^ (UVA3).

These findings align with the results from fresh-cut strawberries, where UV-C irradiation significantly enhanced individual phenolic compounds during storage at 4 °C for seven days. The most abundant compound, ellagic acid, increased by 38.8%, while p-coumaroyl glucose, kaempferol-3-glucoside, and ellagic acid glucoside rose by 123.0%, 60.6%, and 31.75%, respectively. Similarly to TPC trends, phenolic accumulation was greater in UV-C-treated wedges than in controls [47].

Overall, these results confirm that phenolic synthesis is enhanced by increased wound intensity combined with UVA3 irradiation (86.4 KJ m⁻^2^), reinforcing the role of postharvest abiotic stress in optimizing bioactive compound accumulation.

The type of cutting (Ct, *p* < 0.001), UVA radiation dose (UVA-D, *p* < 0.05), and storage temperature (ST, *p* < 0.01) affected the total phenolic compounds quantified using HPLC-PAD (TPC_HPLC_), with a significant quadratic term (UVA-D^2^, *p* < 0.05) (Table S2). Figure 6 shows that cutting treatments alone resulted in minimal variation, with relatively uniform low-to-moderate phenolic abundance. This suggests that the combination of wounding stress and UVA radiation plays a crucial role in enhancing phenolic compound concentration. Notably, TPC_HPLC_ in RM after treatment with UVA radiation with a dose of 86.4 KJ m^−2^ was immediately increased, especially in C3, which showed the highest accumulation (*p* < 0.05), 104%, after UVA3 treatment compared to fresh untreated RM, increasing from 0.81 to 1.65 g Kg^−1^ (Table 5). This value is 80% higher than the TPC_HPLC_ of C3 without UVA-D (0.91 g Kg^−1^). During storage, significant increases (*p* < 0.05) were determined for C3 treated with UVA3 and stored at 20 and 15 °C, the increment in TPC_HPLC_ for UVA3 being significantly higher than the increment in TPC_HPLC_ for UVA6 for C3 stored at 20, 15, and 10 °C (45%, 69%, and 27% higher, respectively).

For UVA6, storage did not promote the accumulation of TPC_HPLC_; however, an immediate increase of 72.6% occurred, with procyanidin B2 as the major compound. The ST of 15 °C and Ct-C3 led to a significant increase (*p* < 0.05) in phenolic compounds by 126% compared to C3 without UVA-D, resulting in a concentration of 2.06 g Kg^−1^ RM, which was the highest concentration of TPC_HPLC_ found in all RM phenolic compound biofortification assays. UVA radiation (320–400 nm) is perceived by plants receptors such as cryptochrome 1 and 2 (CRYs), phototropins 1 and 2 (PHOTs), and three zeitlupe proteins, which play a crucial role in numerous metabolic processes, particularly in the biosynthesis of specific defence compounds [48]. For instance, the accumulation of anthocyanins was reported to increase by 300% in tomatoes when subjected to UVA radiation [49]. In the case of RM, it was determined that UVA-D in synergy with Ct induced the accumulation of procyanidins, which exhibit valuable bioactivities such as cardioprotective, antiviral, anti-cancer, anti-inflammatory, and antioxidant qualities, among others [50,51,52]. Additionally, from an environmental perspective, the application of controlled abiotic stress to agro-industrial residues represents a sustainable approach to valorizing food by-products, reducing waste, and promoting circular economy models [53]. Postharvest treatments that optimize phenolic content contribute to sustainable food processing by enhancing both health benefits and environmental conservation. Food loss remains a global challenge, with industrialized regions losing 15–20% of agricultural production due to strict quality standards, while 15–30% of food is discarded by consumers. In developing regions, losses are higher during postharvest and distribution due to perishability, climate conditions, and market delays [3].

#### 3.2.5. Optimum Experimental Variables for the Combined Biofortification Process Using Wounding and UV-A Radiation Stress

The experimental data obtained were fitted with quadratic polynomial models for each cutting type (Ct) (Supplementary Material S2). Equations (8)–(12) were obtained for the principal responses analyzed (activity of PAL and PPO enzymes, TPC, PACB2, and TPC_HPLC_ concentrations), as shown below.

(8) $$PAL\left[\frac{\Delta A}{h\;mg\;prot}\right]=8.15+27.31\;*\;ST+1.38\;*\;UVA-D-0.02\;*\;ST\;*\;UVA-D-0.69\;*\;{ST}^{2}-0.014\;*\;{UVA-D}^{2}$$

(9) $$PPO\left[\frac{\Delta A}{h\;mg\;prot}\right]=199.39-3.67\;*\;ST-0.76\;*\;UVA-D+0.071\;*\;ST\;*\;UVA-D$$

(10) $$TPC(\frac{g}{Kg})=-42.75+15.01\;*\;ST+1.77\;*\;UVA-D+0.010\;*\;ST\;*\;UVA-D-0.26\;*\;{ST}^{2}-0.011\;*\;{UVA-D}^{2}$$

(11) $$PACB2(\frac{g}{Kg})=6.01+9.83\;*\;ST+1.80\;*\;UVA-D-2.97E^{-3}\;*\;ST\;*\;UVA-D-0.24\;*\;{ST}^{2}-0.010\;*\;{UVA-D}^{2}$$

(12) $${TPC}_{HPLC}(\frac{g}{Kg})=-25.58+10.92\;*\;ST+0.49\;*\;UVA-D-8.58E^{-3}\;*\;ST\;*\;UVA-D-0.21\;*\;{ST}^{2}-3.31E^{-3}\;*\;{UVA-D}^{2}$$

The optimization procedure enabled us to determine the optimal UVA dose, cutting type, and storage temperature to maximize PAL activity, TPC, PACB2, and TPC_HPLC_ concentrations while minimizing PPO activity to avoid the over-oxidation of phenolic compounds of RM. The optimal variables for the combined biofortification process of Granny Smith apple peel using wounding and UVA radiation stress were as follows (Derringer desirability:0.80): UVA-D of 66 KJ m^−2^, Ct 1.5 mm, and ST of 17 °C for a storage time of 56 h.

The validation of the developed models was carried out at the optimal values of the processing variables. The experimental values obtained at the optimal combined biofortification process do not differ significantly (*p* > 0.05) from the predicted values, showing the models’ adequacy. The experimental values at the optimal process conditions were 0.10 ΔA h mg, 0.23 ΔA h mg, 3.80 g AGE/kg, 0.61 g/Kg, 2.02 g/Kg for PAL, PPO, TPC, PACB2, and TPC_HPLC_, respectively.

Previous studies have also reported the benefits of combining different elicitors to enhance the accumulation of phenolic compounds, including UV-B or UV-C and wounding stress or exogenous hormones [11,19,54]. However, using LED UV-A light offers several advantages, like lower risk of manipulation, efficient energy consumption, and lower cost than UV-B and UV-C [45]. Furthermore, the application of wounding stress is a low-cost technique that can be automatized, making it feasible to scale up this type of biofortification procedure for large-scale agro-industrial waste, such as Granny Smith apples peel, yielding compounds of high nutraceutical value.

## 4. Conclusions

This study evaluates the response of Granny Smith apple peel, an agro-industrial by-product, to the application of individual and combined abiotic stresses through wounding and UVA radiation as a tool for biofortification with phenolic compounds. The plant tissue showed a response dependent on the intensity of the wounding stress, and its accumulated systemic response was evident during different storage times and temperatures. The C3 cutting type (1.5 mm wide), the higher stress intensity, and a storage temperature of 15 °C produced significant increases in PAL (1201%), PPO (308%), and the concentration of TPC (108%), PACB2 (22%), and TPC_HPLC_ (118%) at 48 h. The optimal biofortification of apple peel by cutting stress were cutting at 1.5 mm wide (C3) and storing at 17 °C during 56 h. At these conditions, the biofortified apple peel had 2.7 g GAE/Kg, 0.26 g/Kg RM, and 0.90 g/Kg RM for TPC, PACB2, and TPC_HPLC_, respectively. This study demonstrates that wound intensity, storage time, and temperature can modulate the local and systemic response of RM for the production and accumulation of phenolic compounds with high nutraceutical value.

The combined biofortification process using wounding and UV-A radiation stress demonstrated a synergistic activation of the secondary metabolism, enhancing the accumulation of phenolic compounds by up to 174%, with procyanidin B2 (0.78 g/kg) showing the highest increase. These findings support the use of elicitation strategies such as mechanical cutting (1.5 mm), UVA radiation (66 KJ m⁻^2^), and controlled storage (56 h at 17 °C) to maximize phenolic content. From a sustainability perspective, the circular economy approach offers innovative solutions for valorizing apple by-products, transforming waste into high-value products and minimizing environmental impact. The biofortified apple peel can serve as a biofactory of phenolic compounds, offering potential applications in functional food, nutraceuticals, and dietary supplements using safe, low-cost, and efficient technologies. Moreover, the proposed biofortification strategy is scalable, as it utilizes commercially available food processors and energy-efficient UVA irradiation, making it feasible for both industrial and small-scale applications. However, its effectiveness depends on storage conditions, and large-scale implementation requires the further evaluation of sensory attributes, consumer acceptance, and regulatory considerations. Future research should focus on optimizing treatment parameters and assessing bioactive compound stability to enhance their practical applications in food processing.

## Figures and Tables

**Table 1:** Influence of wounding stress and storage time on phenolic compound profile at 20 °C.

Ct	TM (h)	(+)CTQN	PACB2	(-)EPQN	PACT	ACl	Q3G	QP	QHS	K3G	FLN
**C1**	0	0.02 ± 0.002 Ci	0.24 ± 0.005 Aab	0.10 ± 0.003 Ce	0.08 ± 0.02 Af	0.004 ± 0.001 Cg	0.04 ± 0.003 Ci	0.01 ± 0.001 Bb	0.02 ± 0.0003 De	0.06 ± 0.01 Cg	0.0002 ± 0.00003 De
	12	0.06 ± 0.001 Bgh	0.20 ± 0.003 Cde	0.10 ± 0.002 BCde	0.07 ± 0.01 Afgi	0.004 ± 0.0001 BCfg	0.07 ± 0.0003 Bef	0.03 ± 0.0004 Aab	0.04 ± 0.0004 Acd	0.10 ± 0.002 Bdef	0.0006 ± 0.000001 Ccde
	24	0.06 ± 0.002 Bfg	0.21 ± 0.002 BCde	0.11 ± 0.003 ABc	0.08 ± 0.003 Afg	0.005 ± 0.00001 Bef	0.07 ± 0.001 BABe	0.03 ± 0.001 Aab	0.04 ± 0.0001 Abcd	0.10 ± 0.01 Bb-f	0.0009 ± 0.0001 Bbc
	48	0.06 ± 0.001 Bcde	0.22 ± 0.0001 Bcd	0.11 ± 0.01 Ac	0.09 ± 0.007 Af	0.005 ± 0.00002 Bef	0.07 ± 0.0005 ABe	0.03 ± 0.0002 Aab	0.03 ± 0.001 Bd	0.15 ± 0.002 Aa	0.001 ± 0.00002 ABab
	72	0.07 ± 0.001 Aef	0.21 ± 0.003 Cde	0.11 ± 0.01 Ac	0.05 ± 0.01 Ai	0.013 ± 0.0003 Aa	0.08 ± 0.0003 Ade	0.03 ± 0.0001 Aab	0.03 ± 0.0001 Cde	0.15 ± 0.01 Aa	0.0011 ± 0.00002 Aab
**C2**	0	0.01 ± 0.002 Bi	0.21 ± 0.01 ABde	0.08 ± 0.001 Df	0.09 ± 0.002 Cef	0.002 ± 0.00002 Ci	0.05 ± 0.002 Bgi	0.04 ± 0.005 Aa	0.03 ± 0.01 Ade	0.09 ± 0.02 Cfg	0.0003 ± 0.00005 Be
	12	0.05 ± 0.002 Bef	0.20 ± 0.003 Be	0.11 ± 0.0003 Bcd	0.12 ± 0.001 Bde	0.003 ± 0.00002 Cgi	0.09 ± 0.005 Acd	0.04 ± 0.0001 Aab	0.03 ± 0.001 Ad	0.09 ± 0.008 Cfg	0.0009 ± 0.0003 ABbcd
	24	0.05 ± 0.002 Bbcd	0.21 ± 0.005 ABde	0.11 ± 0.001 ABc	0.13 ± 0.001 Aa-d	0.005 ± 0.0006 Bde	0.09 ± 0.001 Acd	0.04 ± 0.0003 Aab	0.03 ± 0.002 Ad	0.11 ± 0.001 ABa-e	0.0011 ± 0.0001 Aab
	48	0.05 ± 0.001 Bb	0.22 ± 0.002 Abcd	0.11 ± 0.001 Ac	0.13 ± 0.001 Abcd	0.006 ± 0.0005 Bd	0.09 ± 0.0005 Ac	0.04 ± 0.0006 Aa	0.04 ± 0.0003 Acd	0.14 ± 0.001 Aabc	0.0011 ± 0.00003 Aab
	72	0.07 ± 0.003 Adef	0.22 ± 0.0001 A	0.10 ± 0.001 Ce	0.05 ± 0.001 Dgi	0.012 ± 0.0004 Ab	0.09 ± 0.0004 Ac	0.04 ± 0.00002 Aa	0.03 ± 0.002 Ad	0.15 ± 0.0003 Aab	0.0011 ± 0.00001 A
**C3**	0	0.03 ± 0.002 Ch	0.17 ± 0.003 Df	0.10 ± 0.003 De	0.13 ± 0.001 Bcd	0.002 ± 0.00003 Di	0.05 ± 0.001 Dfg	0.05 ± 0.0005 Aa	0.04 ± 0.002 Cbcd	0.09 ± 0.01 Cef	0.0004 ± 0.00003 Cde
	12	0.05 ± 0.003 Bb	0.23 ± 0.005 ABabc	0.12 ± 0.001 Bb	0.15 ± 0.002 Aabc	0.003 ± 0.0001 Cgi	0.10 ± 0.0006 Cc	0.02 ± 0.03 Aab	0.03 ± 0.0007 Dde	0.11 ± 0.007 BCc-f	0.0009 ± 0.00004 Bab
	24	0.06 ± 0.001 ABa	0.24 ± 0.001 ABa	0.13 ± 0.002 ABa	0.16 ± 0.002 Aab	0.004 ± 0.0002 Bfg	0.13 ± 0.001 Bb	0.05 ± 0.0001 Aa	0.05 ± 0.0007 Aa	0.12 ± 0.002 ABa-d	0.0011 ± 0.0001 ABab
	48	0.06 ± 0.001 Aa	0.23 ± 0.001 Babc	0.13 ± 0.001 Aa	0.16 ± 0.001 Ba	0.004 ± 0.0002 Bfg	0.14 ± 0.0001 ABab	0.04 ± 0.001 Aa	0.05 ± 0.0004 Babc	0.13 ± 0.005 Abc	0.0012 ± 0.00001 ABab
	72	0.06 ± 0.001 Abc	0.21 ± 0.001 Cde	0.11 ± 0.01 Cc	0.05 ± 0.01 Ci	0.009 ± 0.0001 Ac	0.15 ± 0.01 Aa	0.05 ± 0.0005 Aa	0.05 ± 0.0009 ABab	0.14 ± 0.001 Aab	0.0014 ± 0.00013 Aa

Ct: cutting type. C1: whole apple peel, C2: 5 mm wide, C3: 1.5 mm wide. TM: storage time. (+)CTQN: (+) Catechin; PACB2: Procyanidin B2; (-)EPQN: (-) Epicatechin; PACT: Procyanidin tetramer; ACL: Chlorogenic acid; Q3G: Quercetin-3-o-glucuronide; QPN; Quercetin pentoxide; QHS: Quercetin hexoxide; K3G; Kaempferol-3-o-glucuronide. FLN: Phloretin. Capital letters indicate significant differences (*p* < 0.05) measured using Tukey’s test between storage time for the same cutting type. Lowercase letters indicate significant differences (*p* < 0.05) measured using Tukey’s test between the different storage times and cutting types.

**Table 2:** Influence of wounding stress and storage time on phenolic compound profile at 15 °C.

Ct	TM (h)	(+)CTQN	PACB2	(-)EPQN	PACT	ACl	Q3G	QP	QHS	K3G	FLN
**C1**	0	0.02 ± 0.001 Cgi	0.24 ± 0.004 Acde	0.10 ± 0.003 Aefg	0.08 ± 0.01 Ai	0.004 ± 0.001 Cgi	0.04 ± 0.002 Dl	0.01 ± 0.001 Ci	0.02 ± 0.0003 De	0.06 ± 0.01 Cg	0.0002 ± 0.00002 Eg
	12	0.05 ± 0.003 Ce	0.22 ± 0.004 Bfg	0.11 ± 0.001 Adef	0.09 ± 0.005 Ai	0.006 ± 0.0001 Bef	0.08 ± 0.0003 Bg	0.03 ± 0.0003 Bg	0.02 ± 0.001 Cde	0.1 ± 0.0001 Bef	0.002 ± 0.00007 Bef
	24	0.08 ± 0.0001 Bd	0.22 ± 0.005 ABefg	0.10 ± 0.006 Ag	0.10 ± 0.004 Afgi	0.007 ± 0.0002 Bef	0.07 ± 0.0016 Ci	0.03 ± 0.00001 Afg	0.03 ± 0.001 Bcd	0.14 ± 0.002 Abc	0.0025 ± 0.00003 Abc
	48	0.10 ± 0.003 Aab	0.23 ± 0.002 ABd-g	0.11 ± 0.005 Ad-g	0.12 ± 0.001 Aef	0.011 ± 0.001 Abc	0.08 ± 0.0007 Bg	0.03 ± 0.001 Bg	0.03 ± 0.001 Bcd	0.15 ± 0.0004 Abc	0.001 ± 0.00002 Dbc
	72	0.10 ± 0.002 Aab	0.23 ± 0.009 ABd-g	0.11 ± 0.0007 Ad-g	0.12 ± 0.004 Aefg	0.012 ± 0.0001 Ab	0.09 ± 0.002 Af	0.03 ± 0.001 Afg	0.04 ± 0.001 Abc	0.14 ± 0.002 Abc	0.0015 ± 0.00003 Cbc
**C2**	0	0.01 ± 0.001 Di	0.21 ± 0.01 Bfg	0.08 ± 0.001 Ci	0.09 ± 0.001 Cgi	0.002 ± 0.001 Di	0.05 ± 0.002 Dk	0.04 ± 0.005 ABde	0.03 ± 0.01 Acde	0.09 ± 0.02 Bfg	0.0003 ± 0.00005 Efg
	12	0.06 ± 0.001 Ce	0.22 ± 0.001 Bfg	0.12 ± 0.0003 Ad	0.14 ± 0.003 Bde	0.006 ± 0.0003 Cef	0.10 ± 0.001 Ad	0.04 ± 0.001 Bef	0.04 ± 0.0006 Abc	0.09 ± 0.001 Bfg	0.0014 ± 0.0006 Bfg
	24	0.06 ± 0.001 Cfe	0.23 ± 0.005 Bdef	0.12 ± 0.0005 Ad	0.1 ± 0.006 Abc	0.008 ± 0.001 BCde	0.09 ± 0.001 Cf	0.05 ± 0.001 Abc	0.04 ± 0.0002 Abcd	0.12 ± 0.01 ABc-f	0.0026 ± 0.00001 Ac-f
	48	0.08 ± 0.01 Bcd	0.28 ± 0.005 Aab	0.11 ± 0.01 ABde	0.17 ± 0.0004 Abc	0.009 ± 0.002 Bcd	0.09 ± 0.0002 BCef	0.04 ± 0.0006 ABcde	0.05 ± 0.002 Aab	0.16 ± 0.001 Ab	0.001 ± 0.00002 Cb
	72	0.11 ± 0.002 Aa	0.28 ± 0.008 Aab	0.10 ± 0.00004 Befg	0.16 ± 0.003 Ac	0.015 ± 0.003 Aa	0.10 ± 0.002 ABde	0.04 ± 0.0005 Bef	0.05 ± 0.0007 Aab	0.11 ± 0.001 Bdef	0.0009 ± 0.0004 Ddef
**C3**	0	0.03 ± 0.001 Dg	0.17 ± 0.003 Di	0.10 ± 0.002 Dfg	0.13 ± 0.001 De	0.002 ± 0.00003 Ci	0.05 ± 0.001 Dj	0.05 ± 0.0004 Cbcd	0.04 ± 0.001 Dbcd	0.09 ± 0.006 Cfg	0.0004 ± 0.00004 Dfg
	12	0.06 ± 0.001 Ce	0.26 ± 0.001 Bbc	0.17 ± 0.001 Aa	0.17 ± 0.002 Bbc	0.006 ± 0.0003 Bef	0.12 ± 0.0003 Cc	0.05 ± 0.005 Cbcd	0.05 ± 0.0001 Cab	0.11 ± 0.001 BCc-f	0.0021 ± 0.00001 Bc-f
	24	0.08 ± 0.002 Bdf	0.24 ± 0.004 Ccd	0.14 ± 0.0008 Cb	0.15 ± 0.002 Ccd	0.006 ± 0.00001 Bfg	0.16 ± 0.002 Bb	0.10 ± 0.0005 Aa	0.06 ± 0.001 Ba	0.14 ± 0.001 Bbcd	0.0036 ± 0.00001 Abcd
	48	0.09 ± 0.005 Abc	0.29 ± 0.005 Aa	0.16 ± 0.004 Bb	0.21 ± 0.005 Aa	0.007 ± 0.0003 Bdef	0.18 ± 0.001 Aa	0.05 ± 0.002 Bb	0.06 ± 0.0002 Aa	0.19 ± 0.0004 Aa	0.0018 ± 0.00003 Ba
	72	0.10 ± 0.003 Aab	0.29 ± 0.001 Aa	0.15 ± 0.002 Cbc	0.19 ± 0.008 Bb	0.012 ± 0.0001 Ab	0.16 ± 0.001 Bb	0.04 ± 0.001 Dde	0.05 ± 0.003 Cab	0.13 ± 0.02 Bb-e	0.0014 ± 0.00002 Cb-e

Ct: cutting type. C1: whole apple peel, C2: 5 mm wide, C3: 1.5 mm wide. TM: storage time. (+)CTQN: (+) Catechin; PACB2: Procyanidin B2; (-)EPQN: (-) Epicatechin; PACT: Procyanidin tetramer; ACL: Chlorogenic acid; Q3G: Quercetin-3-o-glucuronide; QPN; Quercetin pentoxide; QHS: Quercetin hexoxide; K3G; Kaempferol-3-o-glucuronide. FLN: Phloretin. Capital letters indicate significant differences (*p* < 0.05) measured using Tukey’s test between storage time for the same cutting type. Lowercase letters indicate significant differences (*p* < 0.05) measured using Tukey’s test between the different storage times and cutting types.

**Table 3:** Influence of wounding stress and storage time on phenolic compound profile at 10 °C.

Ct	TM (h)	(+)CTQN	PACB2	(-)EPQN	PACT	ACl	Q3G	QP	QHS	K3G	FLN
**C1**	0	0.02 ± 0.001 Di	0.24 ± 0.004 BCef	0.10 ± 0.003 Aab	0.08 ± 0.01 Bj	0.004 ± 0.001 Bef	0.04 ± 0.002 Cj	0.01 ± 0.001 Bk	0.02 ± 0.0003 Bg	0.06 ± 0.01 Ch	0.0002 ± 0.00002 Cf
	12	0.04 ± 0.001 Cg	0.23 ± 0.006 Cf	0.10 ± 0.002 Aa	0.11 ± 0.01 AB	0.005 ± 0.0003 ABcd	0.05 ± 0.002 Bi	0.03 ± 0.0005 Aj	0.02 ± 0.0003 Bfg	0.08 ± 0.001 BCefg	0.0026 ± 0.00005 Ac
	24	0.05 ± 0.002 Cf	0.25 ± 0.004 BCde	0.05 ± 0.004 Ch	0.12 ± 0.006 Abe–h	0.005 ± 0.0002 ABbc	0.06 ± 0.002 Ag	0.03 ± 0.0009 Ahi	0.02 ± 0.001 Befg	0.11 ± 0.008 ABbc	0.0003 ± 0.00007 BCef
	48	0.06 ± 0.004 Bde	0.26 ± 0.001 Bcd	0.07 ± 0.004 B	0.12 ± 0.008 ABefg	0.006 ± 0.0004 Aab	0.07 ± 0.0005 Aef	0.03 ± 0.001 Ahi	0.02 ± 0.011 Bfg	0.1 ± 0.0003 BCc-f	0.0005 ± 0.00001 Bef
	72	0.07 ± 0.002 Ab	0.28 ± 0.005 Ab	0.06 ± 0.0002 BCg	0.15 ± 0.01 Abc	0.008 ± 0.0007 Aa	0.06 ± 0.00001 Afg	0.03 ± 0.0005 Ahi	0.03 ± 0.004 Acde	0.15 ± 0.02 Aa	0.0003 ± 0.00001 BCef
**C2**	0	0.01 ± 0.001 Dj	0.21 ± 0.01 ABg	0.08 ± 0.001 Cde	0.09 ± 0.001 Cij	0.002 ± 0.001 Bg	0.05 ± 0.002 Ci	0.04 ± 0.005 Ade	0.03 ± 0.013 Adef	0.09 ± 0.02 Bd-g	0.0003 ± 0.00005 Bef
	12	0.05 ± 0.001 Cf	0.20 ± 0.004 Bg	0.09 ± 0.001 Bc	0.11 ± 0.006 Bghi	0.002 ± 0.002 Bg	0.06 ± 0.002 Bg	0.04 ± 0.001 Afg	0.04 ± 0.001 Aabc	0.08 ± 0.001 Bfg	0.0035 ± 0.0004 Ab
	24	0.06 ± 0.001 Be	0.21 ± 0.008 ABg	0.07 ± 0.001 Df	0.13 ± 0.004 ABdef	0.003 ± 0.002 ABfg	0.07 ± 0.002 ABde	0.04 ± 0.001 Aef	0.04 ± 0.001 Aab	0.15 ± 0.001 Aa	0.0007 ± 0.0003 Bde
	48	0.06 ± 0.001 Bde	0.23 ± 0.003 ABf	0.08 ± 0.0008 Ccd	0.13 ± 0.003 Acde	0.003 ± 0.002 ABef	0.08 ± 0.005 Ab	0.04 ± 0.0003 Ade	0.03 ± 0.0002 Abcd	0.14 ± 0.001 Aa	0.0005 ± 0.00003 Bef
	72	0.09 ± 0.002 Aa	0.24 ± 0.0004 Aef	0.09 ± 0.001 Ab	0.14 ± 0.004 Abcd	0.004 ± 0.002 Ade	0.07 ± 0.001 ABfg	0.03 ± 0.002 Agh	0.03 ± 0.0003 Abcd	0.11 ± 0.001 ABbc	0.0003 ± 0.0001 Bef
**C3**	0	0.03 ± 0.001 Ch	0.17 ± 0.003 Dh	0.10 ± 0.002 Aab	0.13 ± 0.004 BCd-g	0.002 ± 0.00003 Bg	0.05 ± 0.001 Ch	0.05 ± 0.0004 Bbc	0.04 ± 0.001 Aabc	0.09 ± 0.006 BCd-g	0.0004 ± 0.00004 Bef
	12	0.06 ± 0.001 Bde	0.25 ± 0.003 Cde	0.10 ± 0.001 Aa	0.13 ± 0.003 BCcde	0.003 ± 0.00003 ABefg	0.07 ± 0.003 Bde	0.04 ± 0.0007 Bcd	0.04 ± 0.002 Aa	0.10 ± 0.004 Bcde	0.004 ± 0.0002 Aa
	24	0.06 ± 0.0004 Bd	0.26 ± 0.001 BCc	0.07 ± 0.001 Cf	0.15 ± 0.007 ABb	0.003 ± 0.0002 ABef	0.07 ± 0.0007 Bcd	0.05 ± 0.001 Bbc	0.03 ± 0.0004 Bbcd	0.12 ± 0.001 Ab	0.001 ± 0.0002 Bd
	48	0.07 ± 0.004 ABc	0.27 ± 0.004 Bb	0.08 ± 0.001 Bde	0.17 ± 0.007 Aa	0.004 ± 0.001 ABef	0.11 ± 0.003 Aa	0.06 ± 0.002 Aa	0.04 ± 0.001 Aab	0.10 ± 0.002 Bbcd	0.001 ± 0.0001 Bd
	72	0.07 ± 0.0003 Ab	0.34 ± 0.005 Aa	0.07 ± 0.004 BCe	0.10 ± 0.02 Chi	0.004 ± 0.0002 Ade	0.08 ± 0.0005 Bbc	0.05 ± 0.0004 Bb	0.02 ± 0.001 Cfg	0.08 ± 0.001 Cgh	0.0003 ± 0.0002 Bef

Ct: cutting type. C1: whole apple peel, C2: 5 mm wide, C3: 1.5 mm wide. TM: storage time. (+)CTQN: (+) Catechin; PACB2: Procyanidin B2; (-)EPQN: (-) Epicatechin; PACT: Procyanidin tetramer; ACL: Chlorogenic acid; Q3G: Quercetin-3-o-glucuronide; QPN; Quercetin pentoxide; QHS: Quercetin hexoxide; K3G; Kaempferol-3-o-glucuronide. FLN: Phloretin. Capital letters indicate significant differences (*p* < 0.05) measured using Tukey’s test between storage time for the same cutting type. Lowercase letters indicate significant differences (*p* < 0.05) measured using Tukey’s test between the different storage times and cutting types.

**Table 4:** Influence of wounding stress and storage time on phenolic compound profile at 5 °C.

Ct	TM (h)	(+)CTQN	PACB2	(-)EPQN	PACT	ACl	Q3G	QP	QHS	K3G	FLN
**C1**	0	0.02 ± 0.001 Cg	0.24 ± 0.004 Acde	0.10 ± 0.003 Bc	0.08 ± 0.01 Cg	0.004 ± 0.0008 BCb	0.04 ± 0.002 Be	0.01 ± 0.001 Be	0.02 ± 0.001 Cg	0.06 ± 0.012 B	0.0002 ± 0.0002 Bi
	12	0.04 ± 0.001 Aabc	0.22 ± 0.02 ABdef	0.12 ± 0.003 Adef	0.10 ± 0.01 BCf	0.006 ± 0.001 ABab	0.04 ± 0.001 Bde	0.02 ± 0.01 ABde	0.02 ± 0.001 Cfg	0.06 ± 0.001 AB	0.0007 ± 0.0001 Ai
	24	0.04 ± 0.002 ABbcd	0.21 ± 0.01 ABef	0.12 ± 0.004 Aef	0.14 ± 0.001 Bcd	0.007 ± 0.0001 Aab	0.04 ± 0.001 Bde	0.02 ± 0.001 ABd	0.02 ± 0.001 Cfg	0.07 ± 0.004 AB	0.0003 ± 0.00005 ABhi
	48	0.04 ± 0.005 Aab	0.23 ± 0.01 ABcde	0.12 ± 0.004 Acde	0.14 ± 0.01 Bcd	0.007 ± 0.003 Aab	0.05 ± 0.01 Bde	0.02 ± 0.001 ABd	0.02 ± 0.002 Bef	0.07 ± 0.01 AB	0.0004 ± 0.0002 ABf-i
	72	0.03 ± 0.0002 BCfg	0.18 ± 0.02 Bgh	0.05 ± 0.004 Cfg	0.19 ± 0.04 Aa	0.003 ± 0.0004 Cb	0.09 ± 0.01 Aa	0.05 ± 0.01 Aab	0.04 ± 0.01 Ab	0.09 ± 0.007 A	0.0004 ± 0.00002 ABb-e
**C2**	0	0.01 ± 0.001 Dh	0.21 ± 0.01 Aef	0.08 ± 0.001 BCef	0.09 ± 0.02 Dfg	0.002 ± 0.0007 Ab	0.05 ± 0.002 Cde	0.04 ± 0.005 Bbc	0.03 ± 0.001 Aef	0.09 ± 0.02 A	0.0003 ± 0.00005 Bc-f
	12	0.03 ± 0.001 Cfg	0.23 ± 0.02 Acd	0.08 ± 0.002 ABCcd	0.12 ± 0.004 Ce	0.005 ± 0.001 Aab	0.07 ± 0.002 BCc	0.04 ± 0.02 Bc	0.03 ± 0.001 Acde	0.08 ± 0.002 A	0.0007 ± 0.0002 Ad-g
	24	0.04 ± 0.002 Bbcd	0.24 ± 0.01 Acd	0.09 ± 0.001 ABcd	0.12 ± 0.004 Ce	0.005 ± 0.002 Aab	0.09 ± 0.008 Aa	0.04 ± 0.001 Bbc	0.03 ± 0.001 Ade	0.10 ± 0.002 A	0.0005 ± 0.00018 ABabc
	48	0.04 ± 0.001 Aa	0.23 ± 0.01 Acde	0.10 ± 0.001 Acde	0.14 ± 0.007 Bc	0.002 ± 0.0004 Aab	0.08 ± 0.008 ABb	0.05 ± 0.001 Aa	0.03 ± 0.01 Ade	0.10 ± 0.02 A	0.0005 ± 0.00008 ABa-d
	72	0.03 ± 0.0003 Bcde	0.20 ± 0.02 Afg	0.07 ± 0.01 Cfg	0.17 ± 0.004 Ab	0.02 ± 0.0004 Aa	0.09 ± 0.002 Aa	0.04 ± 0.01 Bbc	0.04 ± 0.004 Abcd	0.10 ± 0.004 A	0.0004 ± 0.00008 ABab
**C3**	0	0.03 ± 0.001 Ade	0.17 ± 0.003 Dh	0.10 ± 0.002 Bg	0.13 ± 0.01 Cde	0.002 ± 0.0003 Bb	0.05 ± 0.001 Bd	0.05 ± 0.003 Aabc	0.04 ± 0.001 Bbcd	0.09 ± 0.006 BC	0.0004 ± 0.00004 Ab-e
	12	0.03 ± 0.001 Ade	0.27 ± 0.02 Bb	0.09 ± 0.006 Bb	0.12 ± 0.01 Ce	0.003 ± 0.001 ABb	0.07 ± 0.001 Bc	0.04 ± 0.003 Abc	0.04 ± 0.001 Bb	0.10 ± 0.03 AB	0.0006 ± 0.00009 Aabc
	24	0.04 ± 0.002 A	0.27 ± 0.01 Bb	0.10 ± 0.003 ABb	0.14 ± 0.005 BC	0.004 ± 0.0005 A	0.07 ± 0.003 Bbc	0.04 ± 0.001 Abc	0.04 ± 0.01 ABbc	0.11 ± 0.001 A	0.0006 ± 0.00004 Aa
	48	0.03 ± 0.001 Ade	0.23 ± 0.01 Aa	0.12 ± 0.002 Aa	0.15 ± 0.005 Bc	0.004 ± 0.020 Aab	0.09 ± 0.006 Aa	0.05 ± 0.001 Aab	0.04 ± 0.003 ABb	0.10 ± 0.002 AB	0.0003 ± 0.00008 Aa-d
	72	0.03 ± 0.001 Aef	0.22 ± 0.02 Ccde	0.07 ± 0.004 Ccde	0.20 ± 0.0081 Aa	0.004 ± 0.020 ABb	0.10 ± 0.005 Aa	0.05 ± 0.001 Aabc	0.05 ± 0.003 Aa	0.08 ± 0.003 C	0.0003 ± 0.0001 Ae-h

Ct: cutting type. C1: whole apple peel, C2: 5 mm wide, C3: 1.5 mm wide. TM: storage time. (+)CTQN: (+) Catechin; PACB2: Procyanidin B2; (-)EPQN: (-) Epicatechin; PACT: Procyanidin tetramer; ACL: Chlorogenic acid; Q3G: Quercetin-3-o-glucuronide; QPN; Quercetin pentoxide; QHS: Quercetin hexoxide; K3G; Kaempferol-3-o-glucuronide. FLN: Phloretin. Capital letters indicate significant differences (*p* < 0.05) measured using Tukey’s test between storage time for the same cutting type. Lowercase letters indicate significant differences (*p* < 0.05) measured using Tukey’s test between the different storage times and cutting types.

**Table 5:** Changes in Phenylalanine ammonia lyase (PAL), Polyphenol oxidase (PPO) activity, and total phenolic content (TPC and TPC_HPLC_) at different combined biofortification conditions of Granny Smith apple peel (RM).

Response (%)	Ct	Initial		20 °C		15 °C		10 °C		5 °C	
		UVA3	UVA6	UVA3	UVA6	UVA3	UVA6	UVA3	UVA6	UVA3	UVA6
**PAL**	C1	110.80 aA*	44.03 aAB	149.21 aA*	−58.31 aC	127.88 bA	68.11 aA	113.59 bA*	8.80 aB	108.22 bA*	−52.76 aC
	C2	115.32 aB	148.32 aA	143.67 aA*	−19.47 aBC	162.42 abA*	102.56 aA	114.33 bB*	16.22 abB	149.11 aA*	−60.84 aC
	C3	142.99 aB	148.32 aA	172.82 aAB*	−9.99 aAB	193.34 aA*	38.72 aA	145.02 aAB*	−64.55 bBC	139.68 aB*	−72.94 aC
**PPO**	C1	6.40 aB	−10.21 aC	469.98 aA	112.67 aA	−0.16 cB	63.97 bAB	128.74 bB	−32.69 bC	−8.04 bB	2.82 bBC
	C2	57.81 aC*	26.96 aB	354.87 aA*	138.75 aA	24.66 bC	49.94 bB	130.30 aB	69.74 aB	56.94 aC	29.24 bB
	C3	7.32 aB	1.58 aC	470.60 aA	156.23 aA	108.99 aB	95.23 aB	96.01 abB	115.90 aAB	53.42 aB	92.54 aB
**TPC**	C1	104.64 bA*	21.89 bC	94.19 bA*	58.75 bA	99.15 cA	32.42 bBC	118.86 bA	45.87 aAB	43.16 bB*	19,680 aC
	C2	76.10 bB*	−2.56 bC	70.06 bBC*	41.10 bA	135.42 bA*	20.97 bB	93.49 aBA	−7.38 bC	28.17 aC*	−6.36 aC
	C3	85.08 aB*	12.46 aC	99.84 aB	76.38 aA	174.37 aA*	41.53 aB	103.66 aB*	−17.36 bD	21.28 aC*	−12.26 aD
**TPC_HPLC_**	C1	19.74 bB	66.76 aA	50.97 bA	21.67 a	53.60 cA	37.43 aA	31.58 aAB	22.84 aA	46.65 cA	35.35 aA
	C2	33.64 abB	33.98 aA	44.69 abB*	0.51 aA	62.32 bA	49.12 aA	30.69 aB	−13.32 aA	4.84 abC	−1.75 aA
	C3	80.77 aC	72.59 aA	67.05 aC*	21.98 aBC*	126.15 aD*	57.36 aAB	32.88 aC*	5.53 aC	12.93 aD	7.94 aC

Ct: Wounding type. C1: Whole RM. C2: 5 mm wide. C3: 1.5 mm wide. UVA3: UVA-radiation (86.4 KJ m^−2^). UVA6: UVA-radiation (172.8 KJ m^−2^). Different lowercase letters indicate significant (*p* ≤ 0.05) differences among each wounding type measured using Tukey’s test. Different capital letters indicate significant (*p* ≤ 0.05) differences among different storage temperatures to each UVA-radiation dose measured using Tukey’s test. *: means significant (*p* ≤ 0.05) differences measuring using a *t*-test among different UVA-radiation doses at each storage temperature after 56 h.

## Data Availability

Data is contained within the article and Supplementary Materials.

## Supplementary Materials

The following supporting information can be downloaded at: https://www.mdpi.com/article/10.3390/antiox14030287/s1.
