# Supplementary material for: Combining Abiotic Stresses as a Low-Cost Strategy for Increasing the Phenolic Content in Apple Agro-Industrial By-Products
Source: Antioxidants (Basel). 2025 Feb 27;14(3):287. doi: 10.3390/antiox14030287 (PMC11939634; doi:10.3390/antiox14030287)
Supplement: Supplementary file 1 [file antioxidants-14-00287-s001.zip › antioxidants-3468825-supplementary-done.pdf]

antioxidants

MDPI

Combining Abiotic Stresses as a Low-Cost Strategy for Increasing the Phenolic Content in Apple Agro-Industrial By-Products

Esteban Villamil-Galindo [1,2], Daniel A. Jacobo-Velázquez [3] and Andrea Marcela Piagentini [1,*]

[1] Instituto de Tecnología de Alimentos, Facultad de Ingeniería Química, Universidad Nacional del Litoral, Santa Fe 3000, Argentina; evillamil@santafe-conicet.gov.ar
[2] Consejo Nacional de Investigaciones Científicas y Técnicas (CONICET), Santa Fe 3000, Argentina
[3] Tecnologico de Monterrey, Escuela de Ingeniería y Ciencias, Av. General Ramón Corona 2514, Zapopan 45201, Mexico; djacobov@tec.mx
* Correspondence: ampiagen@fiq.unl.edu.ar

1 Supplementary Material

Table S1. ANOVA for the Phenylalanine Ammonia Lyase (PAL), Polyphenol oxidase (PPO) activity, Total phenolic content (TPC), Total phenolic content by HPLC (TPC_{HPLC}) to RM biofortification assay 1.

Variation Source	DG	PAL	PPO	TPC	PACB2	TPC_{HPLC}
**Ct**	2	***	***	***	*	***
**ST**	1	***	***	***	*	***
**TM**	1	***	***	***	**	**
**Ct x ST**	1	ns	ns	ns	ns	ns
**Ct x TM**	1	ns	ns	*	**	ns
**ST x TM**	1	***	**	ns	ns	ns
**Ct^2**	1	ns	ns	ns	ns	ns
**ST^2**	1	***	**	ns	**	**
**TM^2**	1	***	ns	***	*	***
**Ct x ST x TM**	1	ns	ns	ns	ns	ns
**Ct^2 x ST**	1	ns	ns	ns	ns	ns
**Ct^2 x TM**	1	ns	ns	ns	ns	ns
**Ct x ST^2**	1	ns	ns	ns	ns	ns
**Ct x TM^2**	1	ns	*	ns	ns	ns
**ST^2 x TM**	1	ns	ns	ns	ns	ns
**ST x TM^2**	1	ns	ns	ns	ns	ns
**R^2**	-	0.84	0.62	0.78	0.60	0.70
**Lack of Fit**	53	ns	ns	ns	ns	ns

C: Cutting type, ST: Storage temperature, TM: Storage time, DG: degree of freedom. *: $p \le 0.05$; **: $p \le 0.01$; ***: $p \le 0.001$. ns: $p > 0.05$.

 https://doi.org/10.3390/antiox14030287

Table S2. ANOVA for the Phenylalanine Ammonia Lyase (PAL), Polyphenol oxidase (PPO) activity, Procyanidin B2 (PACB2), Total phenolic content (TPC), Total phenolic content by HPLC (TPC_{HPLC}) to RM biofortification. Assay 2.

Variation Source	DG	PAL	PPO	TPC	PACB2	TPC_{HPLC}
**Ct**	2	**	*	**	**	***
**ST**	1	**	*	**	*	**
**UVA-D**	1	***	*	**	**	*
**Ct x ST**	2	ns	ns	ns	ns	ns
**Ct x UVA-D**	2	ns	ns	ns	*	ns
**ST x UVA-D**	1	ns	*	ns	ns	ns
**Ct^2**	1	ns	ns	ns	ns	ns
**ST^2**	1	**	ns	ns	ns	ns
**UVA-D^2**	1	***	ns	***	***	*
**Ct x ST x UVA-D**	2	ns	ns	ns	ns	ns
**Ct x ST^2**	2	ns	ns	ns	ns	ns
**Ct x UVA-D^2**	2	ns	ns	ns	ns	ns
**ST^2 x UVA-D**	1	ns	ns	ns	ns	ns
**ST x UVA-D^2**	1	ns	ns	ns	ns	ns
**R^2**	-	0.85	0.78	0.75	0.72	0.69
**Lack of Fit**	34	ns	ns	ns	ns	ns

Ct: Cutting type, ST: Storage temperature, TM: Storage time, DG: degree of freedom. *: $p \leq 0.05$; **: $p \leq 0.01$; ***: $p \leq 0.001$. ns: $p > 0.05$.

Table S3. Correlation coefficients for different variables involved in the RM biofortification process assay 1.

	**PAL**	**PPO**	**TPC**	**TPCHPLC**	**(+)CTQN**	**PACB2**	**(-)EPQN**	**PACT**	**ACl**	**Q3G**	**QP**	**QHS**	**K3G**	**FLN**
**PAL**	-													
**PPO**	0.6029***	-												
**TPC**	0.7704***	0.7003***	-											
**TPCHPLC**	0.8033***	0.7194***	0.8734***	-										
**(+)CTQN**	0.7337***	0.3953***	0.6498***	0.7426***	-									
**PACB2**	0.4956***	0.5128***	0.5276***	0.5615***	0.4080***	-								
**(-)EPQN**	0.3185***	NS	0.4541***	0.5369***	0.3360***	NS	-							
**PACT**	0.3685***	0.6711***	0.4802***	0.6642***	0.2824**	0.3125***	0.1990*	-						
**ACl**	0.2304**	0.2868***	0.3361***	0.2934***	0.3571***	NS	NS	NS	-					
**Q3G**	0.7166***	0.5938***	0.8202***	0.8839***	0.5811***	0.3297***	0.5275***	0.5123***	0.2504**	-				
**QP**	0.4869***	0.5058***	0.5100***	0.5390***	NS	NS	NS	0.3964***	NS	0.5485***	-			
**QHS**	0.6036***	0.5588***	0.6842***	0.7741***	0.4529***	0.1883*	0.3837***	0.5352***	NS	0.7877***	0.6613***	-		
**K3G**	0.6697***	0.3877***	0.6138***	0.6986***	0.6826***	0.1928*	0.2553**	0.2107*	0.2947***	0.6060***	0.3423***	0.5486***	-	
**FLN**	0.3846***	NS	0.2489***	0.4110***	0.4199***	NS	0.4120***	NS	NS	0.3988***	0.3314***	0.3523***	0.3117***	-

PAL, Phenylalanine Ammonia Lyase activity; PPO, Polyphenol Oxidase activity; TPC, Total phenolic content; TPCHPLC, Total phenolic content by HPLC. (+) CTQN: (+) Catechin; PACB2: Procyanidin B2; (-)EPQN: (-) Epicatechin; PACT: Procyanidin tetramer; ACL: Chlorogenic acid; Q3G: Quercetin-3-o-glucuronide; QPN; Quercetin pentoxide; QHS: Quercetin hexoxide; K3G; Kaempferol-3-o-glucuronide. FLN: Phloretin. NS: no significative. *$p < 0.05$, **$p < 0.01$, ***$p < 0.001$.

Table S4. Correlation analysis for different responses in studied in RM biofortification process. Assay 2.

Response	(+)CTQN	PACB2	(-)EPQN	PACT	ACl	Q3G	QP	QHS	K3G	FLN	TPC_{HPLC}	PAL	PPO	TPC
**CTQN**	-													
**PACB2**	0.3250*	-												
**EPQN**	NS	0.6236***	-											
**PACT**	0.6076***	0.4928***	0.3875**	-										
**ACl**	NS	0.2896*	0.6926***	NS	-									
**Q3G**	NS	0.4853***	0.6917***	NS	0.8336***	-								
**QP**	NS	0.4154**	0.7195***	NS	0.7878***	0.9143***	-							
**QHS**	NS	NS	NS	NS	NS	NS	NS	-						
**K3G**	0.4095**	0.5471***	NS	0.3411**	NS	0.3079*	0.2798*	0.2798*	-					
**FLN**	0.4250***	0.6758***	0.2815*	0.4588***	NS	NS	NS	NS	0.4278***	-				
**TPC_{HPLC}**	0.4913***	0.8660***	0.7890***	0.5965***	0.5354***	0.7084***	0.2953*	0.2953*	0.5843***	0.5640***	-			
**PAL**	0.4494***	0.4969***	0.4745***	0.5061***	NS	0.2996*	NS	NS	0.2594*	0.5333***	0.5716***	-		
**PPO**	0.5214***	NS	NS	0.3652**	NS	NS	NS	NS	NS	NS	NS	0.2592*	-	
**TPC**	0.4614***	0.5669***	0.5104***	0.4601***	0.2642*	0.3579**	NS	NS	0.2717*	0.7036***	0.6249***	0.7250***	0.3464**	-

PAL, Phenylalanine Ammonia Lyase activity; PPO, Polyphenol Oxidase activity; TPC, Total phenolic content; TPC_{HPLC}, Total phenolic content by HPLC. (+) CTQN: (+) Catechin; PACB2: Procyanidin B2; (-)EPQN: (-) Epicatechin; PACT: Procyanidin tetramer; ACL: Chlorogenic acid; Q3G: Quercetin-3-o-glucuronide; QPN; Quercetin pentoxide; QHS: Quercetin hexoxide; K3G; Kaempferol-3-o-glucuronide. FLN: Phloretin. NS: no significative. *$p < 0.05$, **$p < 0.01$, ***$p < 0.001$.
